# Neighboring Genes Show Correlated Evolution in Gene Expression

**DOI:** 10.1093/molbev/msv053

**Published:** 2015-03-04

**Authors:** Avazeh T. Ghanbarian, Laurence D. Hurst

**Affiliations:** ^1^Department of Biology and Biochemisty, University of Bath, Bath, United Kingdom

**Keywords:** gene expression evolution, gene clustering, sex-biased evolution

## Abstract

When considering the evolution of a gene’s expression profile, we commonly assume that this is unaffected by its genomic neighborhood. This is, however, in contrast to what we know about the lack of autonomy between neighboring genes in gene expression profiles in extant taxa. Indeed, in all eukaryotic genomes genes of similar expression-profile tend to cluster, reflecting chromatin level dynamics. Does it follow that if a gene increases expression in a particular lineage then the genomic neighbors will also increase in their expression or is gene expression evolution autonomous? To address this here we consider evolution of human gene expression since the human-chimp common ancestor, allowing for both variation in estimation of current expression level and error in Bayesian estimation of the ancestral state. We find that in all tissues and both sexes, the change in gene expression of a focal gene on average predicts the change in gene expression of neighbors. The effect is highly pronounced in the immediate vicinity (<100 kb) but extends much further. Sex-specific expression change is also genomically clustered. As genes increasing their expression in humans tend to avoid nuclear lamina domains and be enriched for the gene activator 5-hydroxymethylcytosine, we conclude that, most probably owing to chromatin level control of gene expression, a change in gene expression of one gene likely affects the expression evolution of neighbors, what we term expression piggybacking, an analog of hitchhiking.

## Introduction

Work on the evolution of gene expression has commonly been gene centric, concentrating on, for example, changes in the promoter elements of a given gene (Hammock and Young 2002; Carninci et al. 2006; Tirosh et al. 2006; Wray 2007; Tirosh et al. 2009; Wang and Rekaya 2009; Molineris et al. 2011; Hornung et al. 2012; Rosin et al. 2012; Wittkopp and Kalay 2012; Forrest et al. 2014; Yang et al. 2014). In such a model, changes in the promoter change the expression of the gene controlled by that promoter but nothing else (baring downstream effects of, for example, up- or downregulation of a transcription factor). But are genes autonomous in their evolution in the sense that the change in expression of a focal gene has no effects on its immediate genomic neighbors? In contrast to such an autonomous view of gene expression evolution, when examining profiles of gene expression across chromosomes, it is now evident that in eukaryotes genes of similar expression tend to cluster (Cho et al. 1998; Cohen et al. 2000; Caron et al. 2001; Reik and Walter 2001; Blumenthal et al. 2002; Hurst et al. 2002; Roy et al. 2002; Spellman and Rubin 2002; Birnbaum et al. 2003; Lee and Sonnhammer 2003; Lercher et al. 2003; Versteeg et al. 2003; Khaitovich et al. 2004; Stolc et al. 2004; Williams and Bowles 2004; Denver et al. 2005; Liu et al. 2005; Mijalski et al. 2005; Oliver and Misteli 2005; Singer et al. 2005; Sproul et al. 2005; Lercher and Hurst 2006; Sémon and Duret 2006; Purmann et al. 2007; Ebisuya et al. 2008; Nutzmann and Osbourn 2014). This is seen both at a fine scale and a more gross chromosomal scale (Cohen et al. 2000; Caron et al. 2001; Lercher et al. 2003; Pal and Hurst 2003; Williams and Bowles 2004; Purmann et al. 2007; Michalak 2008; Woo and Li 2011). On a fine scale, neighboring genes tend to be coexpressed more than expected by chance across multiple taxa (Blumenthal et al. 2002; Boutanaev et al. 2002; Roy et al. 2002; Lercher et al. 2003; Fukuoka et al. 2004; Williams and Bowles 2004; Purmann et al. 2007; Davila Lopez et al. 2010), the effect being most pronounced often for genes in a bidirectional orientation, in which promoters sit in close proximity to each other (Cohen et al. 2000; Williams and Bowles 2004; Davila Lopez et al. 2010; Wei et al. 2011; Uesaka et al. 2014). On a more gross scale, genes expressed in most tissues (housekeeping genes) and highly expressed genes tend to cluster in domains corresponding to tens of genes (Caron et al. 2001; Lercher et al. 2002; Versteeg et al. 2003; Weber and Hurst 2011).

Although genes controlled by the same transcription factors are themselves not randomly organized, at least not in yeast (Képès 2003; Janga et al. 2008), in large part broad and narrow span clustering tendencies probably reflect chromatin dynamics rather than shared transcription factors (Grunstein 1997; Cohen et al. 2000; Sémon and Duret 2006; Batada et al. 2007; Li et al. 2007). In yeast, for example, controlling for transcription factor similarity neighboring genes still show striking similarity in coexpression (Batada et al. 2007). Similarly, in mammals, incorporation of transgenes into chromosomes demonstrates that these adopt the expression profile of neighbors within a broad span (Gierman et al. 2007; Symmons et al. 2014). In both yeast and mammals, the upregulation of one gene causes time-lagged ripples of gene expression that correspond to changes in chromatin state (Cohen et al. 2000; Janicki et al. 2004; Ebisuya et al. 2008). In humans these ripple domains are around 100 kb in size (Ebisuya et al. 2008). Whether the fact of clusters of gene expression implies selection for such clusters is unresolved. In yeast, the most highly coexpressed gene pairs tend to be more similar in functionality and more commonly conserved as a pair (Hurst et al. 2002; Poyatos and Hurst 2007). However, results in other lineages are less decisive (Lee and Sonnhammer 2003, 2004; Liao and Zhang 2008; Weber and Hurst 2011).

Here we ask whether genes are autonomous in their expression evolution. To this end we consider RNASeq data for several tissues in male and female primates. Reconstructing the human–chimp ancestral state permits us to estimate the extent of expression change between humans and this ancestor and represent this as a *Z* score that factors in both current variation in expression between replicates (expression or measurement noise) and uncertainty in ancestral state reconstruction. We then consider the extent to which neighboring genes show correlated *Z* scores. Under the null that genes are autonomous in their expression evolution the correlation in *Z* score between neighbors should be zero. In addition, by considering the residuals of the orthogonal regression of *Z* for a gene in a given tissue in males against the same in females we can define the degree of sex bias in expression change. We can thus in turn ask whether this too shows evidence of autonomy.

## Results

### Neighboring Genes Are Correlated in the Expression Change in All Tissues in Both Sexes

So as to gauge what the possible mechanisms might be, we considered several methods to ask whether the expression change of a focal gene (*Z*) is correlated with that of its neighbors. In the first instance we consider for each gene (regardless of which strand they reside on) the nearest neighbor downstream of the focal gene (downstream here is by reference to the published chromosomal strand not to the orientation of the gene), allowing only those instances where the intergene distance is less than 100 kb, this being the estimated size of the ripple effect (Ebisuya et al. 2008), wherein upregulation of one gene causes a time-lagged upregulation of the neighbors (the ripple). In the second instance we consider the correlation between a focal gene and its nearest pair of neighbors, one upstream one downstream, assuming both were within 100 kb (this is comparable to the first method but could be less noisy). In this instance we take the mean *Z* of the neighbors. In the third, we considered for each focal gene the mean *Z* of all neighbors within 100 kb. While the first method might be detecting immediate and local interactions between any given gene pair (e.g., mediated by bidirectional promoters), the latter most likely recovers broader scale chromatin effects. Under the premise that we must be missing the site of expression, we excluded genes with *Z* (prior to modification—see Materials and Methods) of zero owing to lack of expression in a given tissue. In the first and second cases we consider only nonoverlapping genes. For the third case, if the focal gene overlaps any of its adjacent neighbors, it is removed from the analysis; but if there are nonfocal overlapping genes in the neighborhood, they are included.

**Fig. 1.**
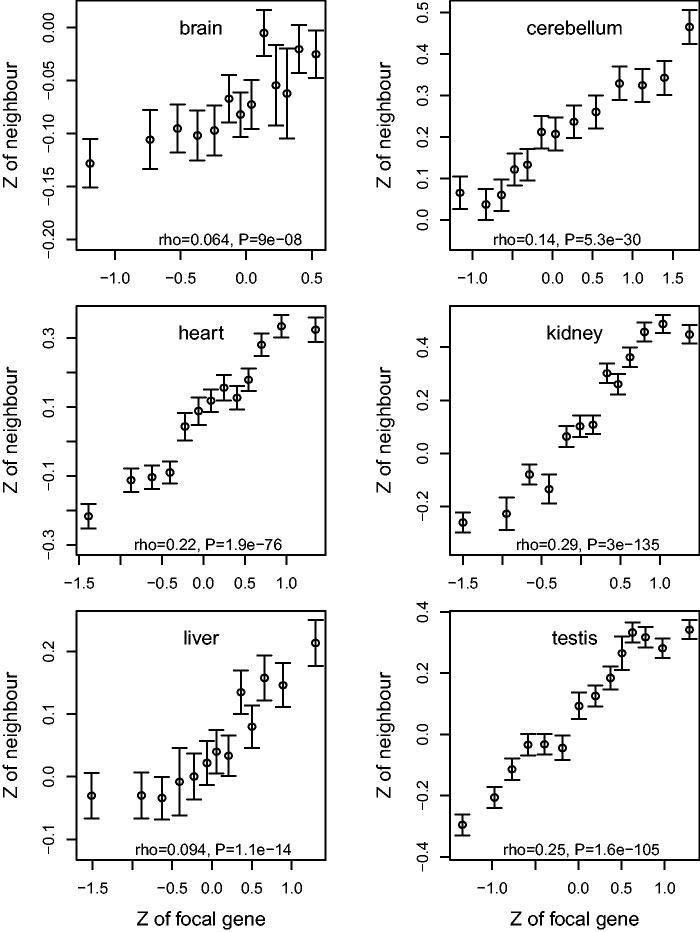
Relationship between *Z* of a focal gene and *Z* of the nearest downstream neighbor for six male tissues. In this instance we consider all genes are nearest downstream neighbors if the distance between the start codons is <100 kb. This slightly contrasts with data in table 1, where the distance is defined as minimum distance between gene bodies. Trends are robust to alternative definitions. Data are split into equal sized bins (of 500 genes) defined after rank ordering with respect to *Z* score of the focal gene. The value on the *X* axis represents the mean *Z* of the genes in that bin. The value of the *Y* axis indicates the mean (±SEM) for the relevant flanking genes. The presented statistics are from Spearman correlation on raw data.

Strikingly we find that for all tissues in both sexes, all analyses report a highly significant positive correlation between *Z* of focal genes and *Z* of neighbors (fig. 1, tables 1–3). The correlation stays highly significant and in positive direction if one is to consider fold change since ancestor instead of *Z* score (supplementary table S1, Supplementary Material online). Note too that our correction of *Z* to a median of zero is here irrelevant as our statistics are based on rank ordering. These results strongly supports the hypothesis that gene evolution is nonautonomous, or at least that it occurs on a cluster-by-cluster basis. We note too that our *Z* scores accord well with the metric to define significantly changed expression employed by Brawand et al (2011) (supplementary fig. S1 and table S2, Supplementary Material online).

**Table 1.** Spearman Correlation between Focal Gene’s *Z* Score and *Z* Score of Its Closest Nonoverlapping Downstream Neighbor.

**Tissue**	**Male *P* Value**	**Male** *ρ*	**Female *P-*Value**	**Female** *ρ*
**Brain**	8.71E−07	0.05504	2.81E−08	0.06247
**Cerebellum**	1.71E−19	0.10246	9.25E−21	0.10539
**Kidney**	3.97E−126	0.26420	3.37E−07	0.05751
**Heart**	4.13E−66	0.19308	7.14E−20	0.10423
**Liver**	5.91E−12	0.07786	NA	NA
**Testis**	6.92E−83	0.21132	NA	NA

Note.—All statistics are significant after Bonferroni testing.

**Table 2.** Spearman Correlation between Focal Gene’s *Z* Score and Mean of Its Closest Nonoverlapping Neighbors on Both Sides.

**Tissue**	**Male *P-*Value**	**Male** *ρ*	**Female *P-*Value**	**Female** *ρ*
**Brain**	2.95E−10	0.08015	8.70E−12	0.08727
**Cerebellum**	1.96E−31	0.15009	1.51E−33	0.15433
**Kidney**	1.44E−155	0.33054	6.07E−10	0.07925
**Heart**	2.03E−86	0.24993	2.16E−28	0.14318
**Liver**	8.86E−17	0.10676	NA	NA
**Testis**	4.43E−118	0.28520	NA	NA

Note.—All statistics are significant after Bonferroni testing.

**Table 3.** Spearman Ranked Correlation of *Z* Score of Focal Gene with Mean *Z* Score of All Its Nonoverlapping Neighboring (within ±100 kb) Genes.

**Tissue**	**Male *P-*value**	**Male** *ρ*	**Female *P-*value**	**Female** *ρ*
**Brain**	7.75E−08	0.04780	6.93E−17	0.07465
**Cerebellum**	8.67E−61	0.14784	1.17E−41	0.12111
**Kidney**	1.32E−274	0.30926	2.81E−15	0.07078
**Heart**	8.82E−160	0.23968	2.07E−44	0.12681
**Liver**	8.51E−26	0.09458	NA	NA
**Testis**	6.27E−187	0.25247	NA	NA

Note.—All statistics are significant after Bonferroni testing.

**Fig. 2.**
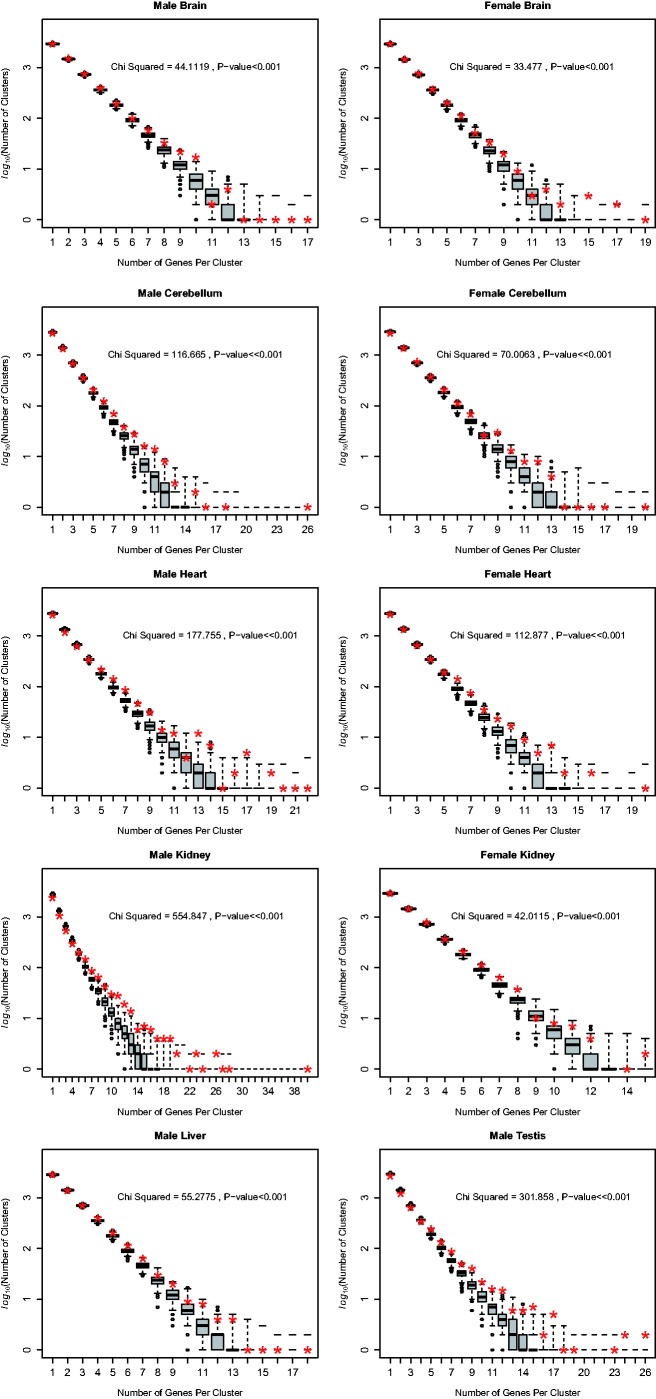
Numbers of clusters of a given size compared to that expected under a random null. Observed number of clusters including certain number of genes is shown by red stars, boxplots show variation across number of clusters in 1,000 random sets.

While the earlier results provide evidence of clustering it does not identify clusters nor does it suggest their dimension. As alternative means to test for clustering and to identify unusually large clusters, we consider the number of switches in *Z* score as one runs along a chromosome. We represent all genes as having a positive, negative, or zero Z score. Those with a zero we consider to be too indecisive to be permitted for this test so are excluded. We then consider, running down each chromosome, the number and lengths of spans with uniform *Z* sign. That is we ask about the size of runs of positive and negative *Z* scores (*Z*+ and *Z*− we then consider as states + and −). To address whether there are fewer but larger runs than expected (clustering) we ask about the number of edges of runs. A series +++–+++ for example has two edges, a + to – switch and a – to + switch. We then compare the observed genomic number of switches to the number expected under a null of random ordering. The null is derived from randomisation of character states (i.e., loci) within each chromosome, thus preserving the absolute number of + and – genes on each chromosome. For all tissues in both sexes, we observe that the observed number of clusters is lower than expected; hence, their length is greater than expected (*P* < 0.0001 in all cases). Put differently, longer runs of uniform expression change are more commonly observed than expected by chance and shorter runs are less common (fig. 2). The largest clusters even by this conservative definition (a single gene of opposite sign breaks a cluster) run to tens of genes. For illustration of some very large clusters, see supplementary figure S2*a* and *b*, Supplementary Material online. This result provides further evidence that our core result, the clustering of genes showing similar change in expression is largely immune to assumption about the precise metric of change, it being seen with *Z* metric (tables 1–3), fold change (supplementary table S1, Supplementary Material online), and digital parametrization (fig. 2).

### Weak Evidence Only That Gene Orientation Is Relevant to Correlated Change in Gene Expression

When considering the correlation between a focal gene and the nearest neighbour, we ignored any effects of orientation between the neighbor and the focal gene. Prior work has suggested that genes in divergent orientation may be particular in the extent of coupling in their expression (Wright et al. 1995; Cho et al. 1998; Cohen et al. 2000; Kruglyak and Tang 2000; Hurst et al. 2002; Trinklein et al. 2004; Williams and Bowles 2004; Woo and Li 2011; Wakano et al. 2012). This may be for no better reason that genes in divergent orientation will have a lower distance between their promoters (Wakano et al. 2012), all else being equal. Genes sharing bidirectional promoters are, under this model, the most highly coupled. Do we then see any effect of the correlation between *Z* scores as a function of orientation?

For every focal gene and its unique nearest downstream neighbour, we consider the two to be in one of three orientations: divergent (<–>), convergent (-><-) and cooriented (->-> or <-<-). For each of the three classes we calculated the Spearman’s *ρ* value for the correlation of *Z* scores between the neighbors, this being repeated for each tissue in each sex (table 4). Very weakly suggestive of a greater coordination of genes in divergent orientation, we find that in 6 of 10 incidences the divergent orientation genes have the highest *ρ* value (these being male liver, brain and testis, and female kidney, heart, and cerebellum). Assuming that the divergent orientation should have the highest *ρ* value one-third of the time, a 6:4 split is not significant (two-tailed, binomial test *P* = 0.094; one-tailed binomial test *P* = 0.076).

**Table 4.** Spearman Correlation between *Z* of Divergent, Convergent, and Cooriented Closest Gene Pairs.

Tissue/Gender	Divergent *P-Value*	Divergent *ρ*	Convergent *P-Value*	Convergent *ρ*	Cooriented *P-Value*	Cooriented *ρ*
Brain/male	*0.000474*	0.07738	0.105396	0.03449	*0.00031*	0.05483
Cerebellum/male	*1.76E−05*	0.09616	*2.27E−07*	0.11123	*7.63E−13*	0.11086
Kidney/male	*1.76E−35*	0.27214	*9.33E−30*	0.23963	*5.83E−78*	0.27992
Heart/male	*4.52E−18*	0.19287	*3.26E−16*	0.17496	*5.52E−42*	0.20693
Liver/male	*8.23E−07*	0.11054	*0.008186*	0.05694	*1.07E−06*	0.07485
Testis/male	*3.47E−30*	0.24745	*1.24E-22*	0.20458	*3.24E-42*	0.20261
Brain/female	*0.003130*	0.06569	*0.000440*	0.07510	*0.00107*	0.05010
Cerebellum/female	*3.01E-10*	0.14002	*0.000271*	0.07796	*1.24E-10*	0.09887
Kidney/female	0.004371	0.06349	0.032372	0.04601	*0.000205*	0.05684
Heart/female	*4.75E-09*	0.13200	*3.77E-06*	0.10040	*2.52E-11*	0.10359

Note.—Results significant after Bonferroni testing are highlighted in italic.

To check whether the three Spearman’s *ρ* values (for each tissue for each sex) differed from *ρ* score of a randomly selected subset of the same size, we performed Monte Carlo randomizations. Each simulation extracted the appropriate but randomly selected number of gene neighbors using the same underlying data (i.e., same tissue, same sex). Each simulation was repeated 10,000 times. The *ρ* score of each random sample was calculated and compared with that observed in the simulants to determine *P* (Materials and Methods). We find that in two incidences (male testis and female cerebellum) genes in divergent orientation have a significantly higher (*P* < 0.05) correlation in the *Z* scores than expected by chance (table 5). The effects are, however, marginal (0.01< *P* < 0.05) and not robust to Bonferroni correction.

**Table 5.** *P*-Values of Monte Carlo Simulations Comparing Spearman’s Correlation *ρ* Score between *Z* Score of Focal Gene and *Z* Score of Its Downstream Neighbor across Divergent, Convergent, and Cooriented Subsets against *ρ* of a Randomly Selected Set of Genes of the Same Size as Those Subsets.

**Tissue**	**Divergent Male *P-*Value**	**Convergent Male *P-*Value**	**Cooriented Male *P-*Value**	**Divergent Female *P-*Value**	**Convergent Female *P-*Value**	**Cooriented Female *P-*Value**
**Brain**	0.12059	0.87421	0.86861	0.37086	0.20748	0.20998
Cerebellum	0.70893	0.40776	0.40526	0.03330	0.91901	0.92151
Kidney	0.41026	0.94881	0.95150	0.36286	0.70813	0.71763
Heart	0.55744	0.86571	0.86821	0.12109	0.68713	0.67243
Liver	0.05359	0.88301	0.88571	NA	NA	NA
Testis	0.03550	0.72293	0.71803	NA	NA	NA

Note.—If the number of genes in divergent orientation, for example, after removing zero *Z* scores in a specific tissue and sex is shown by ts*ND* and Spearman’s correlation’s *ρ* score between those focal genes and their divergent downstream is shown by ts*ρ*. Then *ρ* score of 10,000 random sets of linked gene pairs of *tsND* size, selected from pool of all genes in this study regardless of their orientation, is calculated and compared with *tsρ* in corresponding tissue/gender. If the number of random sets with their *ρ* great or greater than *tsρ* is shown by *M*, Monte Carlo *P-*values are then calculated as (*M*+1)/10,001. No observations are significant after Bonferroni testing.

Prior evidence suggests that bidirectional orientation may have its most profound influence at the sub 1 kb scale (Hurst et al. 2002; Li et al. 2006; Franck et al. 2008), although another study found a marginally lower correlation among divergent genes at 1 kb distance (Takai and Jones 2004). Unfortunately there are few genes in the sample at such proximity. Nonetheless we can repeat the analyses above on this more limited subset. We observe that in five incidences (male brain, male kidney, male liver, female cerebellum, female kidney) divergent orientation records the highest *ρ* value, again not a significant difference (table 6). Weak significance from Monte Carlo simulations is observed in only one case (male liver), again not robust to Bonferroni correction (table 7). We conclude that we see weak, at best, evidence that gene orientation has an influence on the degree of correlated expression change.

**Table 6.** Spearman Correlation between *Z* Score of Focal Gene and *Z* Score of Its Closest Downstream Neighbor across Divergent, Convergent, and Cooriented Closest Gene Pairs Which Are Closer than 1 kb.

**Tissue/Gender**	**Divergent *P-*value**	**Divergent** *ρ*	**Convergent *P-*value**	**Convergent** *ρ*	**Cooriented *P-*value**	**Cooriented** *ρ*
**Brain/male**	0.10085	0.08288	0.81912	0.01280	0.95651	−0.00366
**Cerebellum/male**	0.01006	0.13001	0.01738	0.13288	0.02453	0.15090
**Kidney/male**	*7.07E−16*	0.39189	*1.30E-08*	0.31211	0.00327	0.19567
**Heart/male**	*7.80E-06*	0.22392	*7.79E-09*	0.31661	0.00752	0.17813
**Liver/male**	*0.00044*	0.17669	0.20270	0.07196	0.69872	0.02606
**Testis/male**	*1.02E-11*	0.33586	*1.49E-10*	0.34886	0.04807	0.13197
**Brain/female**	0.36058	0.04629	0.86790	−0.00929	0.43382	0.05267
**Cerebellum/female**	*1.32E-05*	0.21838	0.00461	0.15838	0.05900	0.12635
**Kidney/female**	0.12010	0.07853	0.64196	−0.02613	0.72420	−0.0237
**Heart/female**	0.00250	0.15248	0.00302	0.16604	0.02574	0.14933

Note.—Results significant after Bonferroni testing are highlighted in italic.

**Table 7.** *P*-Values of Monte Carlo Simulation Comparing Spearman’s Correlation *ρ* Score between Focal Gene and Its Downstream Neighbor across Divergent, Convergent, and Coordinated Subsets to a Randomly Selected Subset of the Same Size for Gene Pairs Closer than 1 kb.

**Tissue**	**Divergent Male *P-*Value**	**Convergent Male *P-*Value**	**Cooriented Male *P-*Value**	**Divergent Female *P-*Value**	**Convergent Female *P-*Value**	**Cooriented Female *P-*Value**
**Brain**	0.13399	0.71823	0.72053	0.33787	0.79582	0.79852
**Cerebellum**	0.64264	0.60364	0.59444	0.33907	0.85431	0.84622
**Kidney**	0.17848	0.87671	0.87581	0.07129	0.84862	0.84202
**Heart**	0.91831	0.15938	0.15298	0.78032	0.62664	0.63754
**Liver**	0.02850	0.76262	0.75932	NA	NA	NA
**Testis**	0.57334	0.42326	0.43336	NA	NA	NA

Note.—Monte Carlo simulation’s steps and number of repetition are the same as explained in table 5. No observation is significant after Bonferroni testing.

### Overlapping Genes Are the Most Strongly Positively Correlated in Expression Change

Thus far we excluded from consideration overlapping genes. A priori we might expect these to behave differently, not least because simultaneous expression of both genes might lead to transcriptional interference (Noguchi et al. 1994; Prescott and Proudfoot 2002; Osato et al. 2007). Hence upregulation of one might force downregulation of the other, if only through forcing premature transcriptional termination. Alternatively, upregulation of one might make the chromatin environment of the promoter of the neighbor even more likely to be accessible, so proving an even stronger signal of nonautonomous evolution.

While the original data set (Brawand et al. 2011) was specified as excluding all incidences in which genes overlap within their protein coding sequence, many overlap in their full-length transcript. Examining these we find that the nearest neighbors still show a strong positive correlation in *Z* scores (tables 8 and 9). Indeed, in all cases, the correlation is stronger for the overlapping genes than for the nearest nonoverlapping neighbor. Assuming each sample to be independent, the probability of such agreement is low (binomial test, *P* = 0.002). However, all samples are not independent (male and female expression change correlates—see later). Thus to evaluate whether the strength of this correlation was any different to that expected for any pair of nearest downstream neighbors, we repeatedly extracted from the larger set of nonoverlapping neighbors a random subset of the nearest downstream neighbors. The random subsets had the same number of genes as seen in the overlapping genes’ set. We then asked how often we see a *ρ* value as great or greater than that observed for the overlapping case. Overlapping genes had consistently stronger correlation than the nonoverlapping gene sets in all tissues in both sexes (table 10). These results support the view that close proximity, possibly owing to a greater likelihood of shared chromatin environment, is a more important determinant of coupled gene expression change than is transcriptional interference or gene orientation.

**Table 8.** Spearman Correlation between Focal Gene’s *Z* Scores and *Z* of Its Overlapping Downstream Neighbor on the Opposite Strand.

**Tissue**	**Male *P-*value**	**Male** *ρ*	**Female *P-*value**	**Female** *ρ*
**Brain**	0.00392	0.10783*	0.00368	0.10886*
**Cerebellum**	8.37E−14	0.27613*	8.45E−06	0.16696*
**Kidney**	2.75E−26	0.38295*	0.01655	0.08992*
**Heart**	4.90E−15	0.28986*	1.18E−06	0.18234*
**Liver**	0.00019	0.13979*	NA	NA
**Testis**	<2.2E−16	0.3942*	NA	NA

Note.—Those incidences marked with an asterisk have a higher correlation than seen in the comparable nonoverlapping case (shown in table 1). All observations are significant after Bonferroni testing. As the underlying data are strand-specific transcriptomics, employing overlapping sequence from opposite strands obviates problems with mismapping, causing artifactual signals of high correlation.

**Table 9.** Spearman Correlation between Focal Gene’s *Z* Scores and Mean of Its Closest Up and Downstream Neighbors, at Least One of Which Overlaps the Focal Gene.

**Tissue**	**Male *P-*Value**	**Male** *ρ*	**Female *P-*value**	**Female** *ρ*
**Brain**	0.00013	0.11001*	0.0002	0.10724*
**Cerebellum**	1.18E−24	0.29169*	1.52E−11	0.19365*
**Kidney**	<2.2E−16	0.41596*	0.00126	0.09303*
**Heart**	2.93E−29	0.31778*	4.58E−13	0.20841*
**Liver**	7.60E−07	0.14236*	NA	NA
**Testis**	<2.2E−16	0.4018*	NA	NA

Note.—Those incidences marked with an asterisk have a higher correlation than seen in the comparable nonoverlapping case (shown in table 2). All observations are significant after Bonferroni testing.

**Table 10.** Monte Carlo Simulation of Overlapping Genes’ *Z*.

**Tissue**	**Male *P-*Value**	**Female *P-*Value**
**Brain**	0.005999	0.0095
**Cerebellum**	0.000099	0.003
**Kidney**	0.000099	0.0132
**Heart**	0.000499	0.0004
**Liver**	0.007399	NA
**Testis**	0.000099	NA

Note.—Comparing Spearman correlation’s *ρ* score of overlapping genes against randomly selected set of gene pairs of the same size over 1,000 repetitions. The number of incidents when *ρ* of randomly selected set is equal or higher than *ρ* in overlapping set was counted to calculate empirical *P-*values*.* All observations are significant after Bonferroni testing.

### A Ripple Effect Cannot Explain the Dimensions of the Expression Change Clusters

Although the earlier more extreme correlation in changes at very small distances is potentially consistent with the ripple effect, this same effect suggests that expression clusters should be of ∼100 kb in magnitude (Ebisuya et al. 2008). To estimate physical cluster size, we consider the strength of the correlation between genes in their *Z* score as a function of the distance between them. We consider all focal genes and the correlation between *Z* scores for these genes and the nearest downstream gene at a minimum of *x* base pairs away. By incrementing the minimum distance of *x*, we can then ask at what physical distance on average is *ρ* between the focal genes and nearest “neighbors” is less than the mean ± 1.96 SD of 1,000 randomized null sets.

**Fig. 3.**
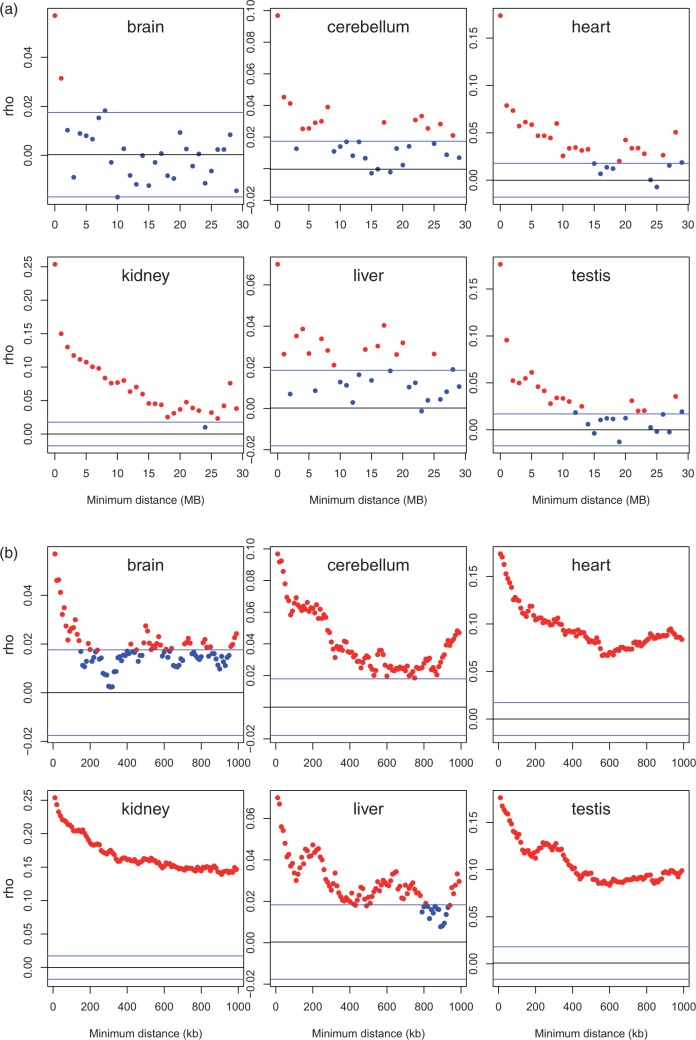
Correlation between *Z* of each focal gene and *Z* of nearest downstream neighbor more than a given minimum physical distance away. (*a*) We plot data considering increments of minimum distance 1 MB at a time up to a maximum of 30 MB. (*b*) We consider 10-kb increments up to a maximum of 1 MB. For each focal gene we extract the nearest neighbor downstream that is at least the distance *x* away, *x* being the units on the *x* axis. From a list of focal and neighbor *Z* scores, we consider then the correlation between these. Correlations significant at the 0.05 level are shown in red, otherwise in blue. The blue horizontal lines indicate 1.96 SD limits determined by randomization (which should in principle correspond with the *P* from Spearman’s *ρ*), with the black line indicating mean of null expectation from randomization (which should be around zero).

For three tissues (heart, kidney, testes), the data appear to be relatively noise free, suggesting the span of local correlation to extend up to tens of megabytes (10–25 MB) (fig. 3*a*). For the remaining three, brain suggests a much more limited domain, while cerebellum and liver are consistent with ∼10 MB span. Looking in more details at trends under 1 MB from the focal genes (fig. 3*b*), we observe that all tissues report the local correlation of *Z* to be most profound under 100 kb, with brain tissue indeed, suggesting this to be the upper limit. The discrepancy between brain and the other tissues might, we suggest be owing to heterogeneity in sampling procedures and intrinsic heterogeneity of brain tissue. A ripple effect (Ebisuya et al. 2008) that extends over ∼100 kb might be able to explain the intensity of the signal at such short range (fig. 3*b*) (notice the nonlinear trends seen in 3*b* and the extent to which the left most data point in 3*a* appears as an outlier). The ripple effect appears, however, to be incompatible with the much longer-range effects as these extend in many cases well beyond the 100 kb limit of the ripple effect.

### Changes in Gene Expression Accord with Lamina Domains and 5-Hydroxymethylcytosine

**Fig. 4.**
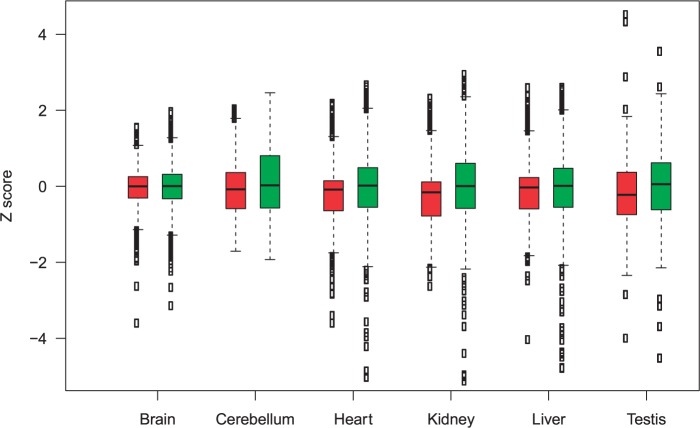
*Z* scores of genes in and out of lamina domains across six tissues. All pairwise comparisons are highly significant (before multitest correction, Mann–Whitney *U* test *P* < 10^−9^ except brain *P* = 4 × 10^−4^). *Z* score of the genes on Lamina domains are shown with boxplots in red and the rest are in green. Genes with very high or very low *Z* are excluded from the plot as outliers to improve presentation but have been included in Mann–Whitney *U* test.

Do the genes changing expression accord with any chromatin signatures? Nuclear compartmentalization and lamina-associated chromatin domains (LADs) in particular have been shown to be involved in regulating genes in Metazoan (Reddy et al. 2008; Van Bortle and Corces 2013). Moreover, recent analysis of gene disregulation in Downs syndrome suggested that LADs represent a level of expression organization in the human genome (Letourneau et al. 2014). LADs have also been shown to associate with low gene expression (Guelen et al. 2008). Hence LADs would provide a good measure for investigating chromatin level regulation’s involvement in evolution of gene expression. Using a high-resolution map of LADs in fibroblast (Guelen et al. 2008), we find that in all six tissues genes residing in putative lamina domains tend to have lower *Z* scores than those not in lamina domains (fig. 4 [before multitest correction, Mann–Whitney *U* test *P* < 10^−9^ except brain *P* = 4 × 10^−^^4^]). Thus increases in expression level tend to be outside of lamina domains.

5-Hydroxymethyl cytosine (hmC) and 5 methylcytosine (mC) are also involved in chromatin level regulation of gene expression through recruiting chromatin modifiers (Mellen et al. 2012; Spruijt et al. 2013). Recent evidence also indicates that gene activity is associated with hmC on the coding strand (Wen et al. 2014). Inactive genes or noncoding strands by contrast tend to be enriched in mC (Dahl et al. 2011). Do we see then any correspondence between hmC, mC (in cortex samples), and *Z*? Employing base pair resolution data (Wen et al. 2014), we indeed observe that *Z* (for brain) is positively correlated with hmC (Spearman correlation: *ρ* = 0.17, *P* < 10^−107^) and negatively correlated with mC (Spearman correlation: *ρ* *=* −0.07, *P* < 10^−18^).

A priori we might expect that genes associated with positive *Z* scores are associated with activating chromatin marks like H3K4me3 (Santos-Rosa et al. 2002; Sims et al. 2003; Martin and Zhang 2005; Greer and Shi 2012). We approach this issue using data from cardiac fibroblast, cardiac myocyte (muscle cells in heart), and astrocytes, chromatin data for which is available. Astrocytes are the most abundant cells in the brain and cerebellum (Tower and Young 1973; Chen and Swanson 2003; Tsai et al. 2012), hence would provide a defendable approximation for histone methylation profile of the whole organ. As expected *Z* score positive genes differ from *Z* score negative ones in H3K4me3 (table 11).

**Table 11.** Number of Positive and Negative *Z* Score Genes Overlapping at Least One H3K4me3 Peak.

**Tissue**	**Number of Genes**	**Number of *Z*+**	**Number of *Z*-**	**AVG (Number of *Z*+ with H3K4me3)**	**Number of Expected *Z*+**	**AVG (Number of *Z*− with H3K4me3)**	Number of **Expected *Z*-**	*χ*^2^ ***P*-Value**
**Astrocytes-cerebellar**	12,418	5,923	6,495	5,108	4,812.38	4,981.5	5,277.12	3.806E−09
**Cardiac fibroblasts**	12,098	5,605	6,493	4,702	4,548.21	5,115	5,268.78	0.00185
**Cardiac myocytes**	12,098	5,605	6,493	4,920.5	4,759.71	5,353	5,513.79	0.00146

Given the earlier result, we might in addition expect that for genes with relatively extreme changes in *Z* the correspondence with H3K4me3 marks should be more pronounced. To address this we consider the subset of genes whose *Z* score is greater than or equal to 1 or less than or equal to −1. Unexpectedly, these genes show no significant difference in their activating histone mark methylation in two instances and only a marginal effect (astrocytes) in one (table 12).

**Table 12.** Number of Highly Positive and Negative *Z* Score Genes Overlapping at Least One H3K4me3 Peak.

**Tissue**	**Number of Genes**	**Number of *Z*+**	**Number of *Z*-**	**AVG (Number of *Z*+ with H3K4me3)**	**Number of Expected *Z*+**	**AVG (Number of *Z*− with H3K4me3)**	**Number of Expected *Z*−**	*χ*^2^ ***P*-Value**
**astrocytes-Cerebellar**	6,164	3,708	2,456	3,206.5	31,32.91	2,001.5	2,075.089	0.03727
**Cardiac fibroblasts**	4,679	2,941	1,738	2,389	2,394.47	1,420.5	1,415.027	0.8544
**Cardiac myocytes**	4,679	2,941	1,738	2,520	2,516.10	1,483	1,486.902	0.8984

Note.—Genes with *Z* score higher than 1 are considered highly positive *Z* and the ones with *Z* score lower than −1 are studied as highly negative *Z.*

The points mentioned earlier shows association of H3K4me3 with elevated expression in human lineage but does not elucidate whether relative gain or depletion of activating histone marks in human compared with other primates are associated with upregulation or downregulation of clusters in human lineage. To address this, we looked for evidence of H3K4me3 peaks with 1.5-fold gain or depletion in human prefrontal neuron samples compared with chimps and macaques (Shulha et al. 2012), in *Z*+ and *Z*− clusters in brain. We found that while *Z*+ clusters are significantly enriched in gained H3K4me3 peaks in both female and male compared with *Z*− clusters, *Z*− clusters are significantly enriched in deplete H3K4me3 peaks compared with *Z*+ clusters only in clusters found in female brain and not males (supplementary tables S4*a* and *b*, Supplementary Material online).

### Genes with Between–Tissue Concordance in Expression Change Are Common and Clustered

Earlier, we have considered each gene’s expression change in each tissue independently. Is it, however, the case that a gene upregulated in one tissue is also upregulated in other tissues or is the effect tissue specific? For those genes showing across-tissue concordance in expression change, do we find that their neighbors also tend to show across tissue concordance? That is, if a gene is up- or down-regulated in all tissues, do the neighbors also show concerted change across all tissues in the same direction as the focal gene?

To ask whether genes tend to show concerted change across all tissues, we start by analysing the six male tissues (as these have multiple replicates making the data more robust). For each gene we then convert the *Z* score into a simple classification (*Z*>0 = +1; *Z*<0 = −1), leaving *Z* = 0 class as is. We then consider the sum of these scores for each gene (*Z* sum). At the limit genes may be downregulated in all tissues compared with the ancestor (*Z* sum = −6) or upregulated in all (*Z* sum = +6). We compare the frequencies of *Z* sum against a null derived from randomizations in which we preserve the sum number of *Z*+, *Z*−, and *Z* = 0 seen in each tissue. We observe a great excess of incidences of concerted change, meaning an excess of more extreme scores (*χ*^2^ = 12,409.04, df = 12, *P* << 0.01; supplementary fig. S3, Supplementary Material online). Indeed, we find 6-fold more genes showing concerted change across all tissues than expected under a null in which the *Z* score in any given tissue is independent of that in any other tissue (table 13). We conclude that there is a strong tendency for change in expression of a given gene to be in the same direction across multiple tissues.

**Table 13.** Observed Number of Concerted Genes Is Higher than Expected.

	**Proportion in:**						**Expected Proportion**	**Expected Number**	**Observed Number**	*χ*^2^	***P-*Value**
	**Brain**	**Cerebellum**	**Heart**	**Kidney**	**Liver**	**Testis**					
***Z*+**	0.4916	0.49996	0.4999	0.4999	0.4999	0.4999	0.015356	200.0482	1216	5159	<<0.001
***Z*-**	0.4804	0.49996	0.4999	0.4999	0.4999	0.4999	0.015006	195.4874	1165	4808	<<0.001

Note.—Concerted genes are either *Z*+ or *Z*− across all six tissues. So the expected number is the mean expectation of the number of concerted genes against a null of independent evolution in all tissues. The total number of genes included in this analysis is 13,027.

Those genes showing concerted evolution across all tissues belong to an eclectic mix of Gene Ontology (GO) terms including sensory perception (for positive concerted *Z* genes) and muscle development regulation (for negative concerted *Z* genes), the logic of which is not transparent to us (supplementary tables S5*a* and *b*, Supplementary Material online).

We can also ask about the expression profile of genes that show high mean *Z* scores. We consider four different metrics of expression, these being expression breadth, peak expression, mean expression level (in the tissues within which the gene is expressed), and expression skew (tau) (for definitions see Materials and Methods). We find that genes with a high mean *Z* score are more broadly expressed (*ρ* = 0.14), more highly expressed (*ρ* = 0.39), have higher maximal expression (*ρ* = 0.38), and have a low degree of skew (i.e., more evenly expressed across tissues) (*ρ* = −0.13) (in all cases *P* < 10^−14^). In many regards, these results are to be expected as high *Z* genes are more likely to be highly expressed genes as *Z* is in part the difference between current and ancestral state and those with the highest current state are likely to be *Z*>0. Consistent with the *Z*+ concerted clusters being housekeeping/highly expressed clusters, in most tissues *Z*+ clusters are shorter and hence denser (although the reverse is observed in clusters in brain), supplementary figure S4 and tables S3*a* and *b*, Supplementary Material online.

To ask whether genes with concerted expression evolution across tissues (all + or all−) are themselves clustered, we ask whether their neighbors are similarly concerted. To this end we identify all genes that show concerted change across all tissues either with positive *Z* or negative *Z* (absolute *Z* sum = 6). We then ask how often we find clusters of such genes (of the same sign). That is, how often do we find two concerted genes of the same sign together, how often we find triplets, *etc*. We compare these numbers to those observed in simulations in which the position of concerted genes is randomized. We find strong evidence that concerted genes clusters occur more than expected by chance (table 14). This suggests a strong principle of clustering of genes that uniformly change expression in the same direction across multiple tissues. Supplementary figure S5, Supplementary Material online, provides some examples.

**Table 14.** Monte Carlo Simulation’s *P-*Value and the Number of Clusters of Concerted Genes of the Same Direction of Evolution of Expression Are Shown by Cluster Size.

*Z* Score Sign of the Cluster	Randomization *P-*Values Per Number of Genes in Clusters/Number of Clusters of This Size				
	2	3	4	5	6
**Positive**	9.999E−05/137	9.999E−05/29	9.999E−05/9	0.0059/2	0.0158/1
**Negative**	9.999E−05/137	9.999E−05/26	9.999E−05/8	1/0	1/0

Note.—Number of *Z*+ and *Z*− concerted genes are kept unchanged, but their order has been randomized, this is repeated for 1,000 iterations. Concerted gene clusters are found, and the number of occurrences of each cluster is compared with observed number of clusters of specific number of concerted genes. If the number is the same or exceeds the observed number of clusters of specific size, Monte Carlo counter is incremented. At the end of the simulation, *P-*value is calculated.

### Tissue-Specific Upregulation Affects Neighbors and Is Common in Cerebellum

If genes that are evolutionarily up- or downregulated across all tissues in humans cluster, do we also see that those showing tissue-specific evolutionary increase tend to sit next to genes showing evolutionary increase in the same tissue? To address this we consider those genes which, in males, show strong (*Z*>1) increase in evolutionary change in one tissue alone, showing zero or negative *Z* in all others. This definition allows recognition of very few genes (170) but suggests the cerebellum to be a hotspot for such change (supplementary table S6*a*, Supplementary Material online). Given the low sample size, we relax the definition to include genes which are *Z*>1 in one and only one tissue, with *Z*<1 in all others. Henceforth, these we will refer to as tissue-specific upregulated (TSU) genes. Analysis of these provide a striking result, namely TSU genes in cerebellum alone are much common than TSU genes in other tissues (supplementary table S6*b*, Supplementary Material online), as indeed are the more strictly defined tissue-specially upregulated genes. We identified 1,230 such genes in cerebellum while only 39 genes show brain-specific upregulation. This we suggest agrees with the recent finding that the cerebellum is a focus of evolution within the primates (Barton and Venditti 2014).

Genes showing tissue-specific upregulation, in contrast to those showing coordinated change across multiple tissues, tend to be in domains of low gene density (the number of genes in ±100 kb of focal gene is low compared with coordinated ones, Mann–Whitney *U* test *P*-value = 1.26 E−43, supplementary fig. S6, Supplementary Material online). This density effect enabled us to compare the local *Z* similarity for the genes with at least one neighbor closer than 100 kb against those whose closest neighbor is further than 100 kb (of which there is an appreciable number). As shown in supplementary table S6*c*, Supplementary Material online, for the genes with a neighbor in 100 kb, the number of focal genes having a *Z*>0 (in the focal tissue) closest neighbor is more than expected by chance (*χ*^2^ = 68, df = 5, *P* < < 0.001). Indeed in all tissues the number of incidences where the nearest neighbor shows upregulation in the tissue of the focal gene is greater than expected, the deviation being significant in four of six tissues. For the genes lacking a close neighbor (supplementary table S6*d*, Supplementary Material online), the trend is mixed but the overall *χ*^2^ statistic is weakly significant (*χ*^2^ = 12.4, df = 5, *P* < 0.05). This, however, is mostly owing to two tissues showing a strong dearth of *Z*+ genes in the vicinity of the TSU genes. That we could not detect an excess of *Z*+ genes outside of 100 kb limit suggests that many tissue-specific change genes are relatively insulated in their effects (compared with what is seen overall), possibly mediated by low gene density.

While earlier we asked merely if the neighbors have an excess of incidence of *Z*>0 in the tissue concerned, we can also ask how many TSU genes have a TSU neighbor (*Z*>1), with that upregulation being in the same tissue (i.e., do we see clusters of tissue-specific upregulation). While no TSU gene has any TSU neighbor in the same tissue in brain and testis, in cerebellum there are 128 genes whose closest downstream neighbor also exhibits cerebellum tissue-specific upregulation. This is not more than expected by chance (one-tailed Monte Carlo simulation keeping the same number of TSU genes in each tissue and randomizing gene order, *P* > 0.05; supplementary table S6*e*, Supplementary Material online). More generally, we see no evidence that TSU genes cluster in any tissue (supplementary table S6*e*, Supplementary Material online) and, through combining individual *P-*values across tissues with Fisher method, we find no overall support for the hypothesis of TSU clustering (*χ*^2^ = 15.84, df = 12, *P*-value > 0.1).

### No Evidence for Unusual Expression Change in the Vicinity of the Human Chromosome 2 Fusion Event

Earlier, we have considered trends en masse. Close scrutiny of some forms of gross chromosomal change suggest that genes neighboring chromosomal disruption sites tend to have altered gene expression (Milot et al. 1996; Dillon et al. 1997; Kleinjan and van Heyningen 1998; Kleinjan and van Heyningen 2005; Harewood and Fraser 2014). Do we see any evidence of this on the broader evolutionary scale? To address this we consider the genes in the vicinity of the human chromosome 2 fusion event.

Human chromosome 2 is fusion of two chromosomes present in the great apes, chimp included (Miller and Reis 1982). The fusion zone is reported to be in the vicinity of 2q13-2q14.1 (Fan et al. 2002). Via the Ensembl web browser (Flicek et al. 2014) under comparative genomic mode, we determined that human gene ENSG00000146556 was in the vicinity of the fusion boundary, its neighbors in chimp being ENSPTRG00000014555 on chromosome 2b in one direction and ENSPTRG00000012388 and ENSPTRG00000012383 on chromosome 2a in the other direction. We then asked whether the mean *Z* for genes in proximity to this site were in any manner unusual. To this end we considered a 1 MB window upstream and downstream of the fusion sites and considered *Z* for all genes within this domain. As expected, in one direction there are relatively few genes, this corresponding to the ancient telomeric end of one of the fusion chromosomes. The mean *Z* score for genes in this window is no different to zero (mean *Z* = 0.002, SD = 0.396), suggesting that this is not a zone associated with either up- or downregulation (supplementary fig. S7, Supplementary Material online).

### Sex-Biased Gene Expression Change Is Clustered

As we have, for several tissues, change in expression data in both males and females, we can ask, for any given gene, whether the change in expression in one sex correlates with that in the other sex. Under a null of no change in the degree of sex bias in expression, such a check also provides an internal consistency check for our mode of analysis and the data. Indeed, as for female tissues we have only one sample, and it might be that data from females are too noisy to be dependable. We find a strong correlation, on a gene-by-gene basis for *Z* in males in given tissue and *Z* in females for the same tissue (table 15). The correlation stays significant when zero *Z* score (after correction) genes are left in (supplementary table S7, Supplementary Material online). This provides support for the hypothesis that the dominant trend in change in gene expression is not sex biased.

**Table 15.** Spearman Correlation between Female and Mean of Male *Z* Scores Per Tissue.

**Tissue**	*ρ*	***P-*Value**
**Brain**	0.52967	<<0.0001
**Cerebellum**	0.32532	<<0.0001
**Heart**	0.45401	<<0.0001
**Kidney**	0.43073	<<0.0001

**Fig. 5.**
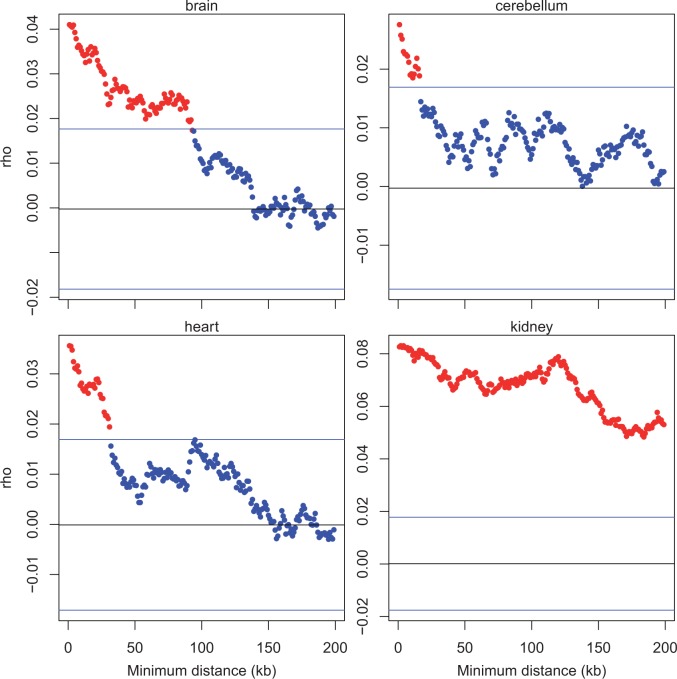
The extent of local correlation in sex-biased expression change for four tissues. Method is the same as that for figure 3, excepting that here we employ standardized residuals of the orthologous regression on *Z* between sexes (rather than *Z*). We consider all focal genes and the correlation between residuals of *Z* scores for these genes and the nearest downstream gene on the same chromosome a minimum of *x* base pairs away. Correlations significant at the 0.05 level are shown in red, otherwise in blue. The blue horizontal lines indicate 1.96 SD limits determined by randomization, with the black line indicating mean of null expectation (which should be around zero).

By considering the standardized residuals from orthogonal regression between the male and female *Z* scores, we can also obtain information on the extent of sex bias in the evolution of gene expression. Note this is not the same as the degree of sex bias, but rather the degree of change in sex bias. We can then ask whether the degree of change in sex bias is also nonautonomous. To this end, we consider the correlations as mentioned earlier. For each focal gene, we consider the correlation between residuals for a focal gene and its nearest downstream neighbor, between the focal gene and its two nearest neighbors (one upstream one down) and between the focal gene and the mean of all neighbors within 100 kb of the focal gene. In all examples we find a significant and positive correlation indicating the sex-biased expression change also occurs in a clustered mode (tables 16–18). In 6 of 8 nearest neighbor comparisons, the effect is more pronounced for overlapping genes. The genomic sizes of the clusters of genes with correlated residuals is varied across tissues, starting with cerebellum and heart clusters below 50 kb, going up to 100 kb in brain and exceeding 200 kb in kidney (fig. 5).

**Table 16.** Spearman Correlation between Sex Bias Standard Residual of Standard Major Axis Estimation between *Z* of Male and Female for a Focal Gene and Standard Residual of Its Nearest Downstream Neighbor.

**Tissue**	**Nonoverlapping *P-*Value**	**Nonoverlapping** *ρ*	**Overlapping *P-*Value**	**Overlapping** *ρ*
**Brain**	*0.00018*	0.03995	*0.00325*	0.10407
**Cerebellum**	0.03109	0.02304	*9.10E−06*	0.15636
**Heart**	*1.42E−05*	0.04638	*8.04E−05*	0.13913
**Kidney**	*6.95E−19*	0.09465	0.01206	0.08883

Note.—Incidences significant after Bonferroni testing are shown in italic.

**Table 17.** Spearman Correlation between Standard Residual of Standard Major Axis Estimation between *Z* of Male and Female for a Focal Gene and Mean Standard Residual of Its Two Nearest Neighbors.

**Tissue**	**Nonoverlapping *P-*Value**	**Nonoverlapping** *ρ*	**Overlapping *P-*Value**	**Overlapping** *ρ*
**Brain**	*1.46E−05*	0.05452	*0.00281*	0.07649
**Cerebellum**	0.01433	0.03082	*6.07E−07*	0.12738
**Heart**	*4.50E−07*	0.06346	*3.05E−08*	0.14127
**Kidney**	*7.02E−23*	0.12348	*4.32E−06*	0.11740

Note.—Incidences significant after Bonferroni testing are shown in italic.

**Table 18.** Spearman Correlation between Standard Residual of Standard Major Axis Estimation between *Z* of Male and Female of the Focal Gene and the Mean of Standard Residual of All Its Neighbors within 100 kb of the Focal Gene.

**Tissue**	**Spearman *P-*Value**	**Spearman** *ρ*
**Brain**	4.00E−08	0.04817
**Cerebellum**	0.00848	0.02310
**Kidney**	1.71E−39	0.11504
**Heart**	1.87E−05	0.03755

Note.—All incidences are significant after Bonferroni testing.

These results support the hypothesis that the extent of change in sex bias is also genomically regionalized. This is further supported by the finding that when we score residuals as positive or negative states, we again find fewer switches in state than expected by chance, implying clustering (*P* from randomisation, brain *P* = 0.0009; cerebellum *P* = 0.01; heart *P* = 0.007; kidney *P* = 0.001).

The earlier analysis ignores those instances where *Z* is zero (before median correction) for a gene in either sex. This may be biasing results as the genes with *Z* = 0 in one sex, but not the other, are sex biased in their change of expression. This makes little difference to results (supplementary tables S8*a*–*c*, Supplementary Material online).

### No Evidence That the X Chromosome Is Enriched for Genes Changing Sex Bias

With the same data we can also ask whether another form of clustering is seen, i.e., chromosomal scale clustering. According to Rice’s hypothesis (Rice 1984) the X chromosome should be a hotspot for sex-biased gene expression change. He postulates that genes with sexually antagonistic fitness effects can be more likely to spread if on a sex chromosome. The spread of such alleles creates the context for the spread of modifiers that limit the expression of the deleterious allele in the sex in which the effect is deleterious, i.e., modifiers of sex-specific change in expression. Hence sex biased gene expression change is expected to be more pronounced on the X chromosome than on autosomes. This can mean both the evolution toward male-biased and female-biased gene expression.

Given that we have no strong prior on the direction of sex-biased change on the X, we consider for all genes the modulus of the degree of sex-biased change. We then ask whether these values are different for X than for autosomes. We find no evidence for a difference (Mann–Whitney *U* test, brain *P*-value = 0.4906; cerebellum *P*-value = 0.8944; heart *P*-value = 0.9374; kidney *P*-value = 0.7523). In addition we can ask about the 5% of genes with the most extreme change in sex bias (the 5% with the highest modulus of residual score). Are these more commonly found on the X chromosome? We find no evidence to support this proposition either (supplementary table S9, Supplementary Material online). We conclude that we see no evidence that the X chromosome is a hotspot for sex-biased gene expression change. However, if instead we consider the change in expression of genes in the testis, we do find that X-linked genes show a different median *Z* compared with autosomal genes. Considering only those genes with expression >0 in the ancestor, the median *Z* for X-linked genes is 0.15, while for autosomes it is −0.012 (Mann–Whiney *U* test, *P* = 0.00023). In no other tissue is the median *Z* on the X greater than the median *Z* on the autosomes.

## Discussion

Here we have presented evidence that gene expression change, at least in humans, occurs on a cluster-by-cluster basis, such that the expression change of any given focal gene predicts the expression change of genes in its vicinity in any given tissue. The result is insensitive to the metric of expression change. Moreover, many genes show coordinated changes in expression across multiple tissues and in the same tissue in different sexes. Genes that show coordinated expression changes across multiple tissues tend to sit next to other genes showing similar coordination. This suggests that a dominant mode of expression change evolution may be nothing more than a switch of a chromosomal block to a state of permanently open (or predominantly closed) chromatin in multiple tissues (or open/closed longer in multiple tissues), thereby causing increases or decreases in expression of spans of genes in all circumstances.

Gene density effects we suggest might in addition also be relevant. If much of the expression change is owing to local chromatin modification, we might expect that domains of high gene density are more coordinated in their expression change, simply because the chances that a local change to one gene might affect another would be greater. Such a model is consistent with our finding that genes showing tissue-specific upregulation and that have no gene neighbor within 100 kb do not affect expression of their nearest (over 100 kb) neighbor, while other genes in high density domains do. If upregulation of one gene in a zone of high gene density affects the neighbors whose upregulation affects the neighbors on and so forth, this might in turn generate self-propagating domains of expression change. It is notable then that genes showing increased expression across multiple tissues tend to be in domains of high gene density.

Why gene expression for the focal gene changes is unclear, although we found no evidence for a coupling with chromosomal alternations (i.e., in the chromosome 2 fusion event). While the precise mechanisms of nonautonomous evolution are unclear, the form of the curves relating genomic distance to correlation in *Z* score, suggest much more profound effects in immediate vicinity, a conclusion supported by the stronger correlations seen for overlapping genes. We suggest that there may thus be more than one mechanism at play. Perhaps in the immediate vicinity of a gene, expression of one gene directly impacts the expression of its neighbors (cf. the ripple effect [Ebisuya et al. 2008]), while over broader spans (>100 kb), a more generic chromatin opening/closing and self-propagation mechanism (Batada et al. 2007; Gierman et al. 2007) may be more relevant. Either way, our results suggest that a promoter-focused concentration on the causes of expression change (Tirosh et al. 2009; Rosin et al. 2012; Wittkopp and Kalay 2012; Yang et al. 2014) is likely to provide too restricted a view of expression change viewed more globally, at least within primates.

While we detected expression change clusters defined on an intrachromosomal scale, which for the most part is not predicted by population genetical theory, we did not observe a form of clustering that we had expected from such theory. Rice’s theory (Rice 1984) would suggest that X-linked genes should be prone to changes in sex-biased gene expression; however, we did not detect this for expression in tissues present in both sexes. One possible explanation for this might be that the tissues examined may not be those most likely to be subject to the strongest sex-biased gene expression. Indeed, testes show a large increase in *Z* for X-linked genes compared with autosomal genes, potentially compatible with Rice’s model (note this is not change in degree of sex bias as there is no female testicular expression to compare it with). The data thus accord with a model in which for nonsex-specific tissues the degree of sex-biased change in gene expression is a largely neutral process and thus outside of the domain of Rice’s hypothesis.

More generally, given the extent to which one gene’s expression change affects that of the neighbors, it is simplest to suppose as a null model that much of the expression change we observe is neutral and what might be called expression “piggybacking.” That is to say, the upregulation of one gene may be selectively favored but, because its upregulation increases the chances that the neighbors are upregulated, the spread through the population of the focal heritable expression change causes expression divergence (from the ancestral state) of near neighbors of that focal gene. The expression change of the neighbours need not be the focus of selection but rather a necessary consequence of the change to the focal gene.

Expression piggybacking may be considered an analog of genetic hitchhiking, in so much as it suggests correlated changes at genomically neighboring sites. Piggybacking is different, however, in so much as it does not require linkage disequilibrium between alleles at closely linked sites. Indeed, in piggybacking there need only be one allele affecting the expression of the focal gene while the neighboring genes can, in principle, be genetically uniform across the population. Nonetheless, the flanking genes will change, over evolutionary time, their expression profile, piggybacking on the heritable expression change at the focal allele. Alternatively put, estimation of the net selective impact, if any, of any mutation affecting the expression of any given gene, needs also to factor in the effects this focal expression change has on the expression of neighbors as well. Our data are broadly consistent with expression piggybacking, possibly largely selectively neutral, being a fundamental cause of expression divergence in primates.

## Materials and Methods

### Estimation of Z Scores

Gene expression data were obtained from Brawand et al (2011). We used expression values reported in NormalizedRPKM_ConstitutiveAlignedExons_Primate1to1Orthologues.txt and extracted loci and strand information from Human_Ensembl57_TopHat_UniqueReads.txt also provided in the supplementary materials of the relevant paper. This provides RPKM figures for 13,027 genes in six tissues across five primate species. To determine the change in gene expression between current levels in humans and that seen in the human–chimp common ancestor we employed BayesTraits (Pagel et al. 2004). The assumed phylogeny and branch lengths are the same as those employed by Brawand et al. (2011).

BayesTraits was run in the following manner. Normalized RPKM, as provided by Brawand et al. (2011), were passed to BayesTraits as measures of gene expression. For each gene, mean of normalized RPKM values across different individuals in Human was calculated separately for male and female samples. Also if more than one male or female sample is available in any of the tissues in chimpanzee or any of the outgroups, their mean is computed and passed to BayesTraits, otherwise a single expression value was used. To find the estimated gene expression level in the ancestor of human and chimpanzee, for each gene in each tissue, BayesTraits program was run twice, first to build the estimated gene expression tree for males and second for female samples. Each time, the primate phylogenic tree and means of normalized RPKM of the gene in human and also its orthologous genes in chimpanzee and three primate outgroups (gorilla, orangutan, and macaque), in corresponding gender, are passed to BayesTraits, to build the estimated gene expression model. BayesTraits employs Markov chain Monte Carlo and maximum likelihood to find the posterior distribution of this model and estimate the level of expression in this tree’s middle nodes (Pagel et al. 2004). Through examination of the convergence trends of the BayesTraits output, we considered that the final 10% of BayesTraits estimates would be robust. From this sample we estimate both the mean (*E*_a_) and variance (*V*_a_) in the estimation of the human–chimp ancestral state. Relaxation of the 10% cutoff makes no important difference to results (data not shown).

These simulations were run independently for each gene, for each tissue in each sex. If the mean expression of given gene, in given tissue in a given sex is *E*_current_, or *E*_c_ in abbreviated form, and its variance is *V*_c_, if estimable, while that for the ancestral condition is *E*_a_ and *V*_a_, then we can define the degree of expression divergence in human lineage from human–chimp ancestor as a *Z* score:

$$Z=\frac{E_{c}-E_{a}}{\sqrt{V_{c}+V_{a}}}$$

This metric compares the extent of difference between mean current expression level and ancestral level, scaled by the degree of variation both in current estimates (expression noise or measurement error) and the degree of uncertainty in the ancestral state’s estimation. A positive *Z* implies an increase in gene expression since the ancestor. In part the defense for our metric is the same as the defense for any application of a *Z* score, namely it measures difference in standard deviation units. That is, a gene with largely variable expression across individuals or high fluctuation and uncertainty in estimation of expression in ancestor would have a lower *Z* score compared to a gene with similar but steadier level of current expression and/or one with similar but more stable estimation of ancestral level of expression. However, another part of the defense is that in our model, inspired by the ripple hypothesis, increased opening of chromatin can lead to increased spurious expression. Our supposition is that this might cause an approximately constant absolute increase in the amount of transcription in all neighbors a given distance away, not an increase proportional to the current level (as measured by fold change). Nonetheless to examine the possibility that results might be contingent on metric we also consider 1) a digital representation (increase or decrease since ancestor) and 2) fold change. Note too that we are not concerned with whether our metric calls significance in gene expression change as most of the gene expression in our model is neutral drift owing to ripple effects. Rather, we wish to present a quantitative variable that captures the absolute amount of expression change factored in standard deviation units.

For each tissue in each sex we assume that the median expression change must be zero. This is equivalent to assuming an absence of net increase or decrease in overall expression levels. This required a minor adjustment of *Z* scores for all genes in all tissues. If the median *Z* in any given tissue in a given sex is *M*, then we defined modified *Z* as *Z*_mod_ = *Z* – *M*. This forces all tissues to have a modified median of zero and as many genes increasing expression as decreasing (this being approximately equivalent to an assumption that the net transciptome size is no different; hence, for every gene increasing expression there should be one decreasing expression). All analyses were performed on *Z*_mod_. Henceforth, we shall refer to *Z*, for convenience, where *Z*_mod_ is what we are employing. In practice the correction makes little or no difference as 1) the correction is usually very small and 2) many of our statistics are rank order based and so unaffected by the modification. We note that our method has the advantage that it largely eliminates any RNAseq amplication biases (e.g., owing to GC content) from affecting our metric of expression change. This is because nucleotide content is almost unchanged between human and chimp, and hence any bias in amplification of a given transcript is likely to affect human and chimp equally. By considering only the change from the ancestor we thus exclude amplification biases from derivation of *Z*. As evidence for this, the mean correlation, across all tissues, between *Z* and the change in GC between human and chimp is indistinguishable from zero.

### Chromatin Data

For a few human cell lines, ChIP-seq histone methylation data produced by University of Washington is available through ENCODE’s portal (Bernstein et al. 2012; Gerstein et al. 2012; Rosenbloom et al. 2012). We could approximate whole tissue histone methylations profile by matching the most abundant cell lines in heart and cerebellum to three of the cell lines available in ENCODE. Among many cell types composing heart, Cardiac fibroblast and cardiac myocyte (muscle cells in heart) are consequently mostly abundant ones. Furthermore, astrocytes are the most numerous cell type in the central nervous system (Chen and Swanson 2003; Tsai et al. 2012). Hence, HAc, an astrocytes-cerebellar cell line, was used to approximate histone methylation profile in cerebellum (Tower and Young 1973).

To do the histone methylation analysis, H3K4me3 peak data were downloaded as an activating histone mark (Santos-Rosa et al. 2002; Sims et al. 2003; Martin and Zhang 2005; Greer and Shi 2012) for above cell lines. Then *Z* score positive and negative genes overlapping one or more H3K4me3 peak(s) were found using Bedtools (Quinlan and Hall 2010). Due to the histone mark protocol used in ENCODE, each experiment was repeated twice and peak data are reported separately for each repetition. So here we report the average number of *Z* score positive or negative genes overlapping one or more peaks across these two repeated peak data sets.

We also compared *Z* score positive and negative clusters with regard to gain and depletion of H3K4me3 peaks in humans compared with chimps and macaques. To do this, we took 885 H3K4me3 peaks which were shown to have 1.5-fold higher human-specific gain in human samples compared with macaque and chimpanzee samples as shown by Shulha (2012). Intersect command from bedtools was then used to find the clusters overlapping a gained H3K4me3 peak. An ad hoc script was used to count the number of *Z*+ and *Z*− clusters with at least one gained peak. Similarly we also compared the number of *Z*+ and *Z*− clusters with evidence for at least one H3K4me3 depleted peak using 177 H3K4me3 peaks with human-specific depletion which had at least 1.5-fold lower tag density in human samples compared with chimps and macaques as shown by Shulha (2012).

### GO Analysis

Is there a functional link between the genes that show the same sign of expression change across all tissues (concerted genes)? Is there a functional clue to link the genes with elevated changed expression across all tissues? To determine this, the concerted genes (same profile of change across all tissues) are divided into two sets:first the ones with elevated expression in human lineage compared with human–chimp ancestor across all tissues in male samples and second the ones with reduced expression than the estimated expression in the ancestor. We just used male sample tissues for this analysis as there are more repeats available for these, also as shown, their expression is more stable and less noisy. Doing this, we found 1,244 concerted *Z* score positive genes and 1,053 concerted negative ones. Then GO term enrichment analysis was performed on these two sets, using GOrilla (Eden et al. 2009), to find the enriched GO functions and processes.

### Expression Measures

To address the correlates of *Z* we also ask about a series of expression measures, these being breadth, mean rate, peak rate, and tau. For a gene to be considered as being expressed in a given tissue in a given species we required that the mean across replicates for that tissue to be more than at least 2 RPKM. If it was less than 2, it was set to zero for that tissue. Breadth is defined as the proportion of tissues within which a gene is expressed. To prevent nonindependence between rate and breadth, we defined rate as the mean rate of expression of that gene across all tissue within which it is expressed (i.e., at rate > 2). Peak rate is the maximum expression level considered across all tissues. Tau is a measure of skew in expression and is defined as:

$$\tau =\frac{\sum_{j=1}^{n}(1-\frac{\text{log}(e_{j})}{\text{log}(e_{\text{max}})})}{n-1}$$

where there are *n* tissues, the expression in any one being *e*_j_ and the maximal for that gene across all tissues is *e*_max_. A gene with very highly skewed expression (very high in only one tissue) take a high value of tau (limit approaching 1) while those expressed uniformly take a low value (limit zero).

### hmC and mC Assays

Base resolution map of hydromethylome in prefrontal cortex has been produced by Wen et al. (2014). First shown in Bacteriophage, hmC is able to turn genes on or off (Wyatt and Cohen 1952; Dahl et al. 2011). Wen et al. (2014) has recently shown 10-fold increase in hmC in adult prefrontal cortex compared with fetal. Also, hmC correlates positively with gene expression while mC correlates negatively with gene expression (Colquitt et al. 2013; Wen et al. 2014). Furthermore, there is disparity between hmC and mC enrichment on sense and antisense strands, hmC being enriched on sense and mC on antisense strands (Peric-Hupkes et al. 2010). To find out if they correspond with change in gene expression, we took hmC and mC percentages as reported by Wen et al (2014) and calculated how they correlated with *Z* scores of genes in brain.

### Lamina Domain Assignment

LADs originally produced by Guelen et al (2008) using Lung fibroblast cell line, are available through UCSC’s table browser for hg19. Intersect command from bedtools (Quinlan and Hall 2010) was used to find the genes overlapping these domains. For this analysis, genes with zero *Z* scores (prior to modification) are not removed due to expectation of the genes on LAD domains to be very lowly, if at all, expressed. Then *Z* of genes on and off LAD domains were compared using Mann–Whitney *U* test and also Brunner Munzel test, to correct for robustness to the form of distributions.

### Statistics

Where appropriate statistics were performed in R, many analyses were performed using Monte Carlo simulations. In these incidences, if *N* is the number of observations as extreme or more extreme as observed and *M* is the number of simulants, then the unbiased estimator of the type I error rate (what may be regarded as an empirical *P*) is:

$$P=\;\frac{N+1}{M+1}.$$

## Supplementary Material

Supplementary figures S1–S7 and tables S1–S9 are available at *Molecular Biology and Evolution* online (http://www.mbe.oxfordjournals.org/).

Supplementary Data
