# Supplementary material for: Neighboring Genes Show Correlated Evolution in Gene Expression
Source: Mol Biol Evol. 2015 Mar 4;32(7):1748–66. doi: 10.1093/molbev/msv053 (PMC4476153; doi:10.1093/molbev/msv053)
Supplement: Supplementary Data [file supp_32_7_1748__index.html]

Neighboring genes show correlated evolution in gene expression — Neighboring Genes Show Correlated Evolution in Gene Expression — Neighboring Genes Show Correlated Evolution in Gene Expression — Supplementary Data 

# Neighboring Genes Show Correlated Evolution in Gene Expression

## Supplementary Data

files

**Files in this Data Supplement:**

- Supplementary Data - pdf file
- Supplementary Data - pdf file
