# Supplementary material for: Neighboring Genes Show Correlated Evolution in Gene Expression
Source: Mol Biol Evol. 2015 Mar 4;32(7):1748–66. doi: 10.1093/molbev/msv053 (PMC4476153; doi:10.1093/molbev/msv053)

**Fig S1. The genes Brawand et al found to have changed their expression significantly in human also have high Z score.** Genes listed with significant lineage-specific expression switches in human in Brawand et al. are divided in two groups: Brawand's significant genes which are Z score positive (in blue) and the ones with negative Z scores (in yellow). These two subsets are then plotted against the rest of the genes, shown in green, across different tissues. There was no Z score negative gene in 4 genes Brawand found to have significantly changed their expression in human brain. Please also note that genes found to have extremely high or low Z score have been removed from this plot to improve clarity of comparison between our method and Brawand et al's method.

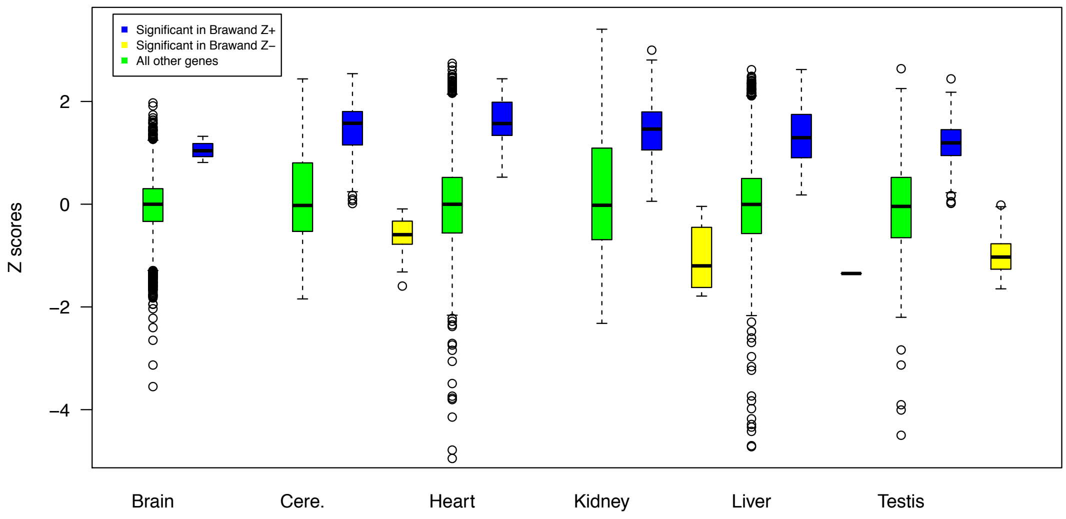

# Fig S2a. Examples of large positive Z clusters

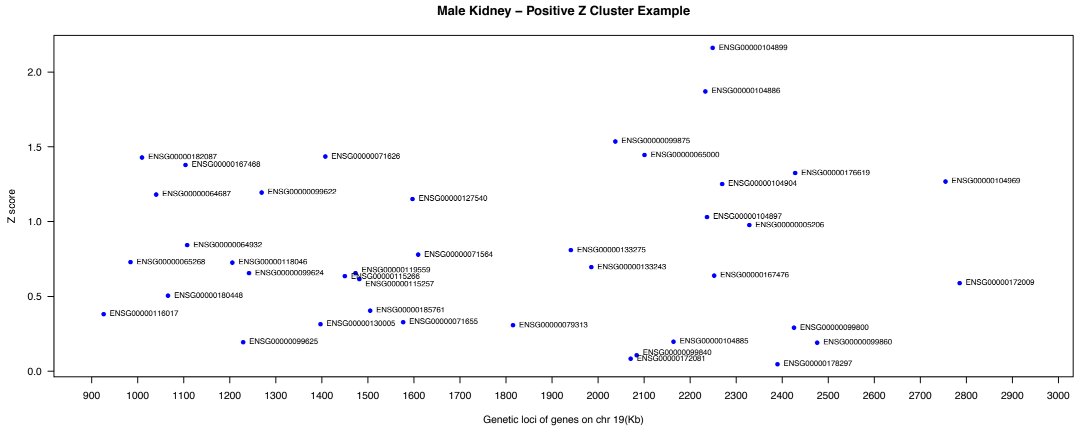


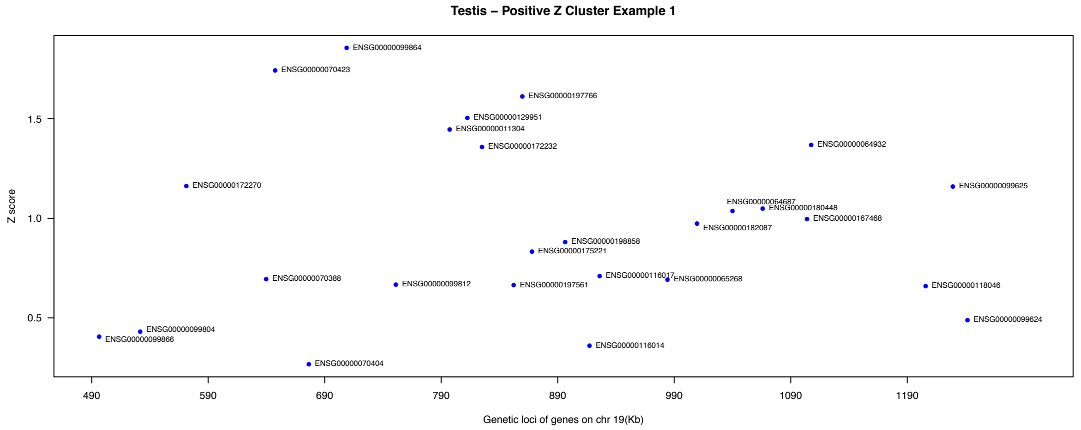


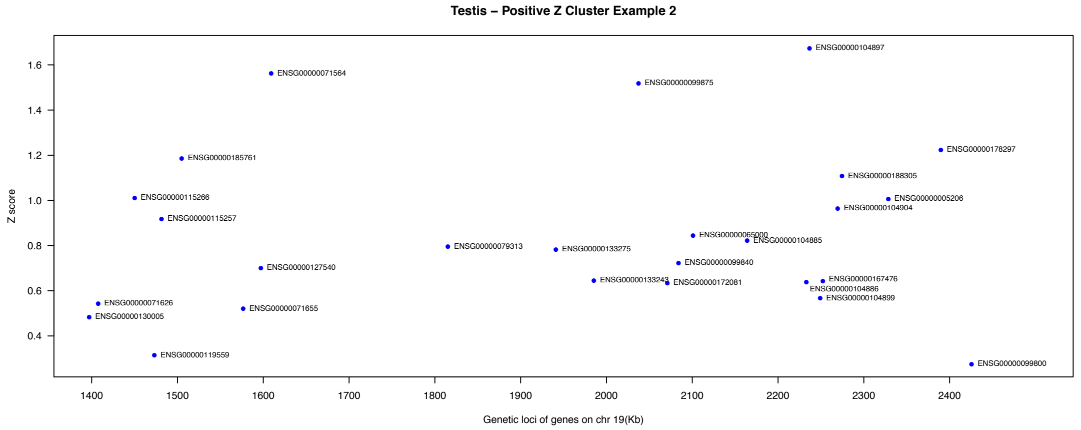

# Fig S2b. Examples of large negative Z clusters

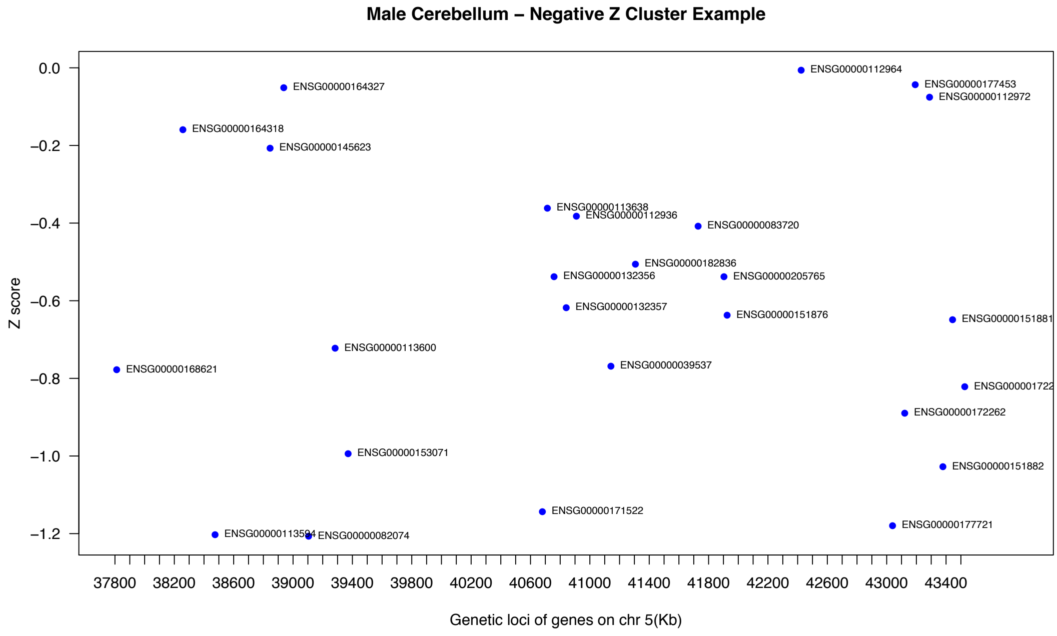


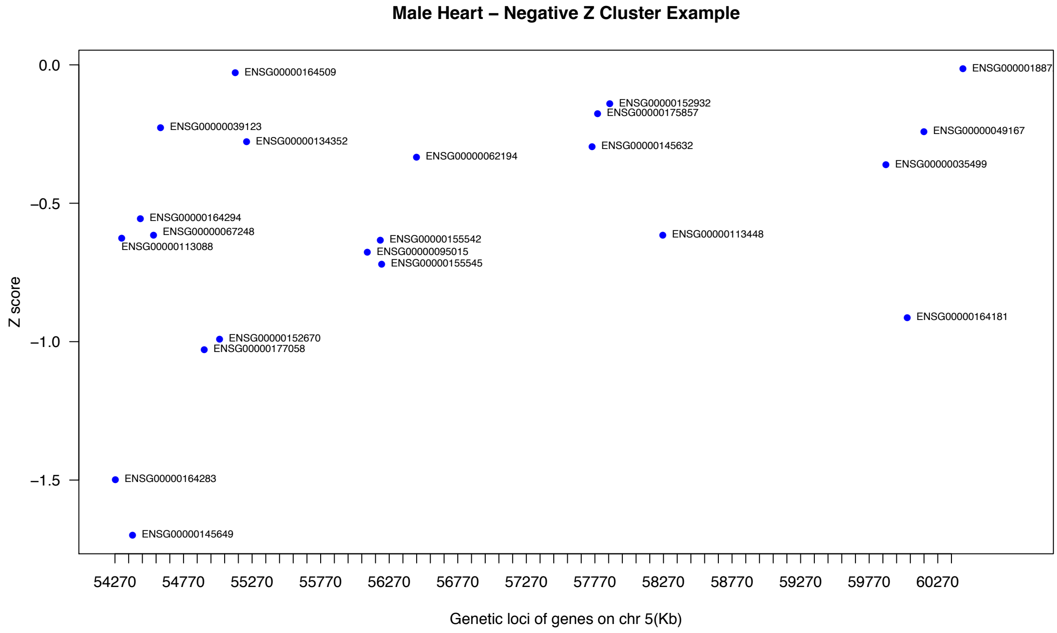

**Fig S3. Genes showing increase in expression across all tissues or decrease in expression across all tissues are more common than expected by chance**. The sign of Z score indicates the direction of change in gene expression across time (from common ancestor to human). One may represent Z positive genes with +1 and Z negative with -1, keeping zero Z scores unchanged, and calculate for each gene their sum across 6 male tissues. Genes with sums equal to +6 (or -6) have gone up (or down) across all 6 tissues. The number of concerted up-regulated genes is shown by the right-most bar in histogram and the number of concerted down-regulated genes is shown by the left-most bar. Null expectations are shown in blue. Null is derived from randomizations in which the number of Z+, Z- and Z=0 genes are kept the same as observed, but the gene order is randomised across tissues. Errors bars are +/-SD from 1000 simulations.

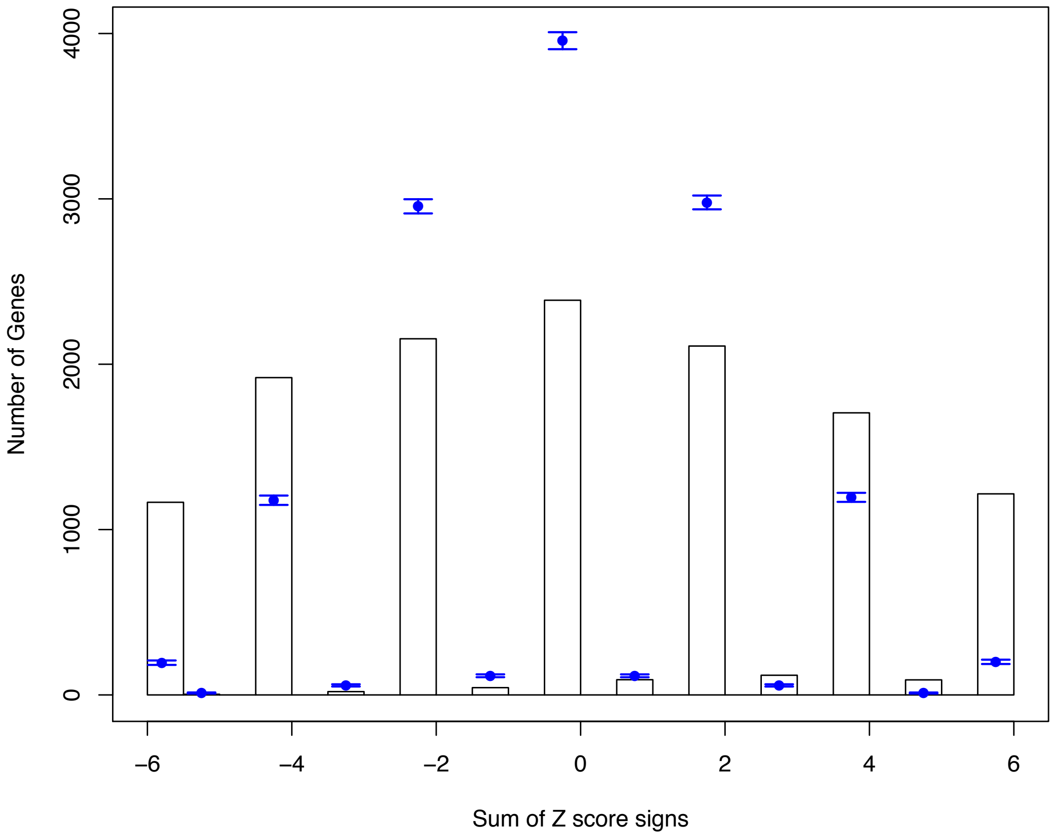

**Fig S4. Positive Z score clusters are denser in most tissues except in brain.** Positive Z score clusters are shown in green and negative ones in red. The effect is most pronounced in male kidney.

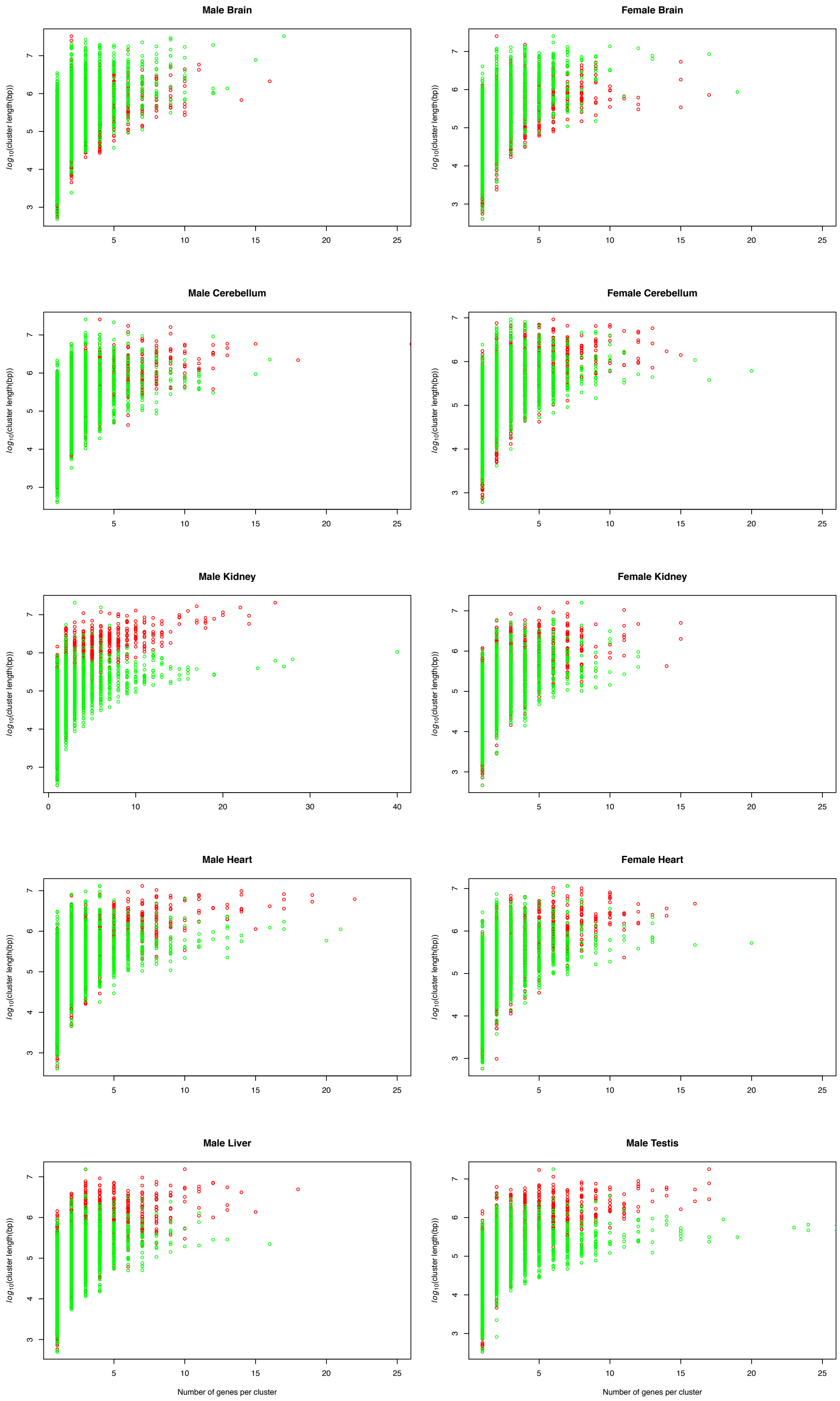

## Fig S5. The longest concerted Z score positive cluster based on expression profile in males

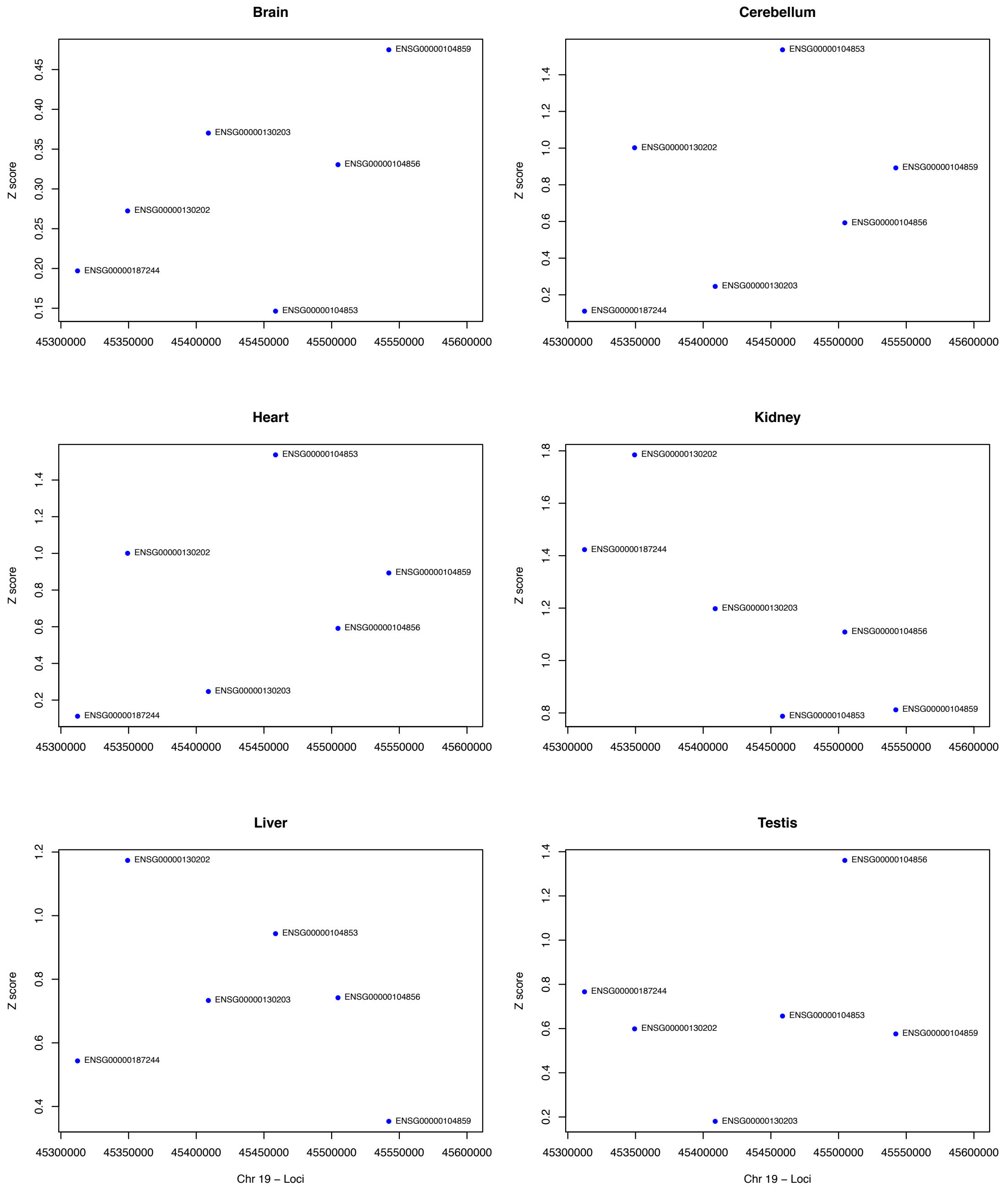

**Fig S6. Tissue-specific up-regulated genes tend to be in domains of low gene density compared to genes with concerted change in expression across all 6 tissues.** Number of genes in ±100Kb window around the tissue-specific up-regulated and concerted genes are shown below. There is a significant difference between number of genes in ±100Kb of focal genes is tissue-specific genes compared to concerted ones, Mann-Whitney U test *P-value* = 1.26e-43.

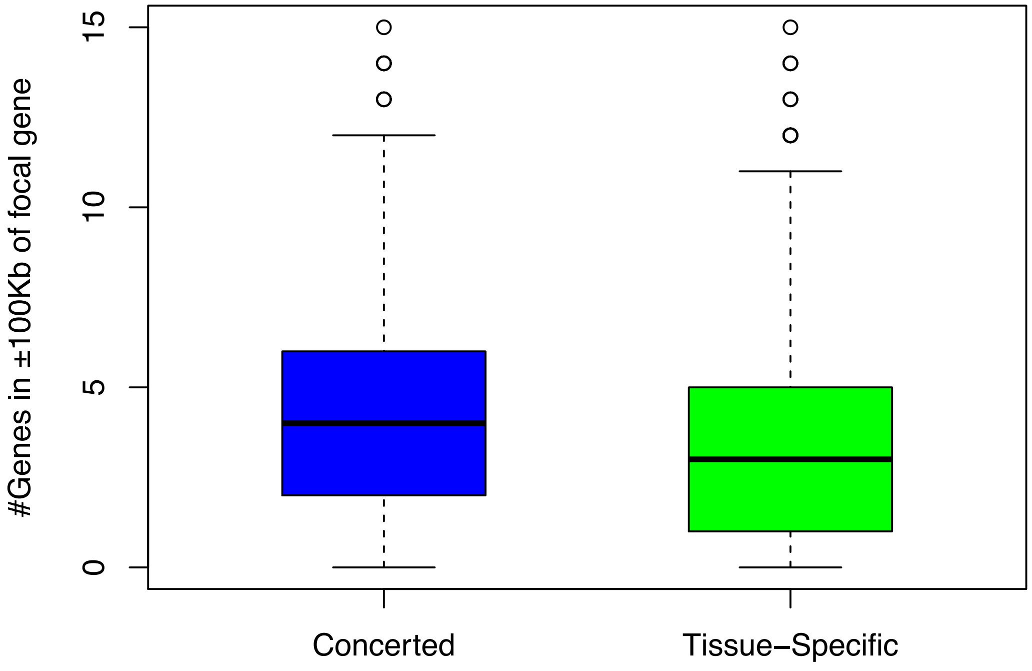

**Fig S7. Mean of Z scores of genes across 6 tissues in the vicinity of fusion point on human chromosome 2.** Means are averages across all tissues. Error bars indicate SEM. The vertical line indicates the approximate location of the fusion zone.

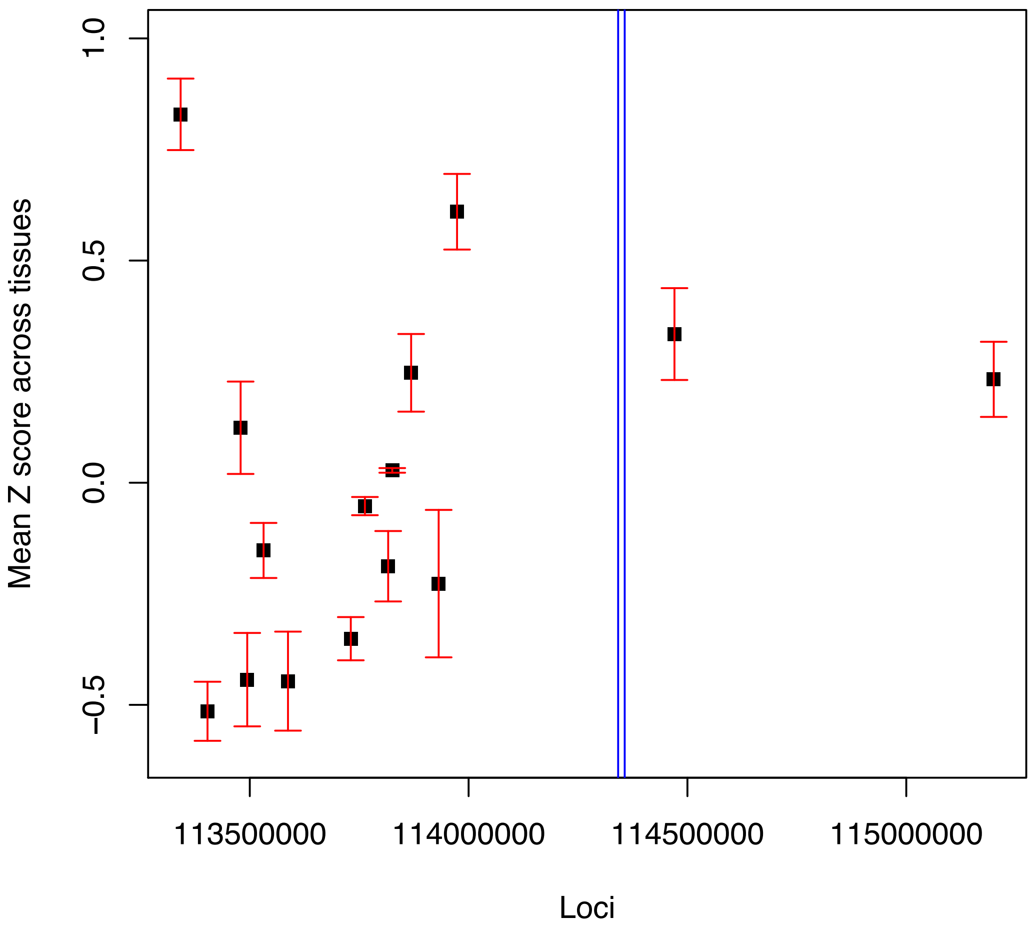

Supplement: Supplementary Data [file supp_msv053_Supplementary_Figures_1-7_220115.pdf]
