# Supplementary material for: Neighboring Genes Show Correlated Evolution in Gene Expression
Source: Mol Biol Evol. 2015 Mar 4;32(7):1748–66. doi: 10.1093/molbev/msv053 (PMC4476153; doi:10.1093/molbev/msv053)
Supplement: Supplementary Data [file supp_msv053_Supplementary_tables_1-9_230115.pdf]

Table S1a. Spearman correlation between focal gene's fold change and fold change of its closest non-overlapping downstream neighbor. Fold change is defined as mean of current level of expression in human divided by mean of estimated expression in ancestor All statistics are significant after Bonferroni testing.

Tissue	Male *P-value*	Male *Rho*	Female *P-value*	Female *Rho*
**Brain**	9.61E-10	0.06680	1.71E-10	0.07036
**Cerebellum**	5.15E-22	0.10794	7.50E-31	0.12778
**Kidney**	1.29E-107	0.23983	1.57E-08	0.06260
**Heart**	2.29E-43	0.15370	3.10E-22	0.11004
**Liver**	3.41E-16	0.09092	NA	NA
**Testis**	7.16E-92	0.21573	NA	NA

Table S1b. Spearman correlation between focal gene's fold change and mean fold change of its closest non-overlapping neighbors on both sides. All statistics are significant after Bonferroni testing.

Tissue	Male *P-value*	Male *Rho*	Female *P-value*	Female *Rho*
**Brain**	5.12E-18	0.09341	2.49E-19	0.09756
**Cerebellum**	3.88E-38	0.14116	6.95E-57	0.17230
**Kidney**	6.99E-149	0.27710	3.74E-17	0.09157
**Heart**	2.48E-59	0.17661	1.72E-35	0.13684
**Liver**	1.24E-23	0.10929	NA	NA
**Testis**	5.20E-145	0.26955	NA	NA

Table S1c. Spearman ranked correlation of fold change of focal gene with mean of fold change of all its non-overlapping neighboring (within ±100Kb) genes. All statistics are significant after Bonferroni testing.

Tissue	Male *P-value*	Male *Rho*	Female *P-value*	Female *Rho*
**Brain**	3.51E-09	0.05254	3.43E-10	0.05617
**Cerebellum**	4.81E-55	0.14054	2.47E-59	0.14527
**Kidney**	9.39E-251	0.29607	9.68E-26	0.09391
**Heart**	4.99E-109	0.19835	4.44E-42	0.12333
**Liver**	2.30E-53	0.13815	NA	NA
**Testis**	5.32E-178	0.24651	NA	NA

Table S2. Concordance between genes called as of changed expression by Z score method and the method of Brawand et al. Mann-Whitney test comparing Z scores of the genes shown by Brawand et al to have significantly shifted their expression compare to the rest of the genes shows significant difference between Z score of the two groups. The number of genes reported as significant in Brawand et al. is very low in some tissues.

**Tissue**	**Male *P-value***	**Female *P-value***	**Number of genes in Brawand's significant genes list**
**Brain**	0.00068	0.00177	4
**Cerebellum**	9.87E-88	4.71E-94	268
**Kidney**	1.42E-42	8.04E-75	207
**Heart**	6.01E-07	1.16E-06	10
**Liver**	3.42E-36	NA	80
**Testis**	4.83E-154	NA	567

Table S3a. GO processes enriched in concerted Z score positive genes across tissues in male samples

GO term	Description	*P-value*
GO:0050909	Sensory perception of taste	1.97E-5
GO:0007606	Sensory perception of chemical stimulus	1.41E-4
GO:0009968	Negative regulation of signal transduction	1.48E-4
GO:0048585	Negative regulation of response to stimulus	7.82E-4
GO:0060041	Retina development in camera-type eye	8.78E-4
GO:0017038	Protein import	9.03E-4

Table S3b. GO processes enriched in concerted Z score negative genes across tissues in male samples

GO term	Description	*P-value*
GO:0016202	Regulation of striated muscle tissue development	4.99E-4
GO:1901861	Regulation of muscle tissue development	4.99E-4
GO:0048634	Regulation of muscle organ development	4.99E-4

Table S4a. In Brain Z+ clusters, in comparison to Z- clusters, are significantly enriched in gained H3K4me3 peaks in human compared to chimps and macaques in both males and females.

Tissue	#Clusters	#Positives Cluster	#Negatives Negative	#Positives cluster gained at least one H3K4me3	#Expected positives clusters gained	#Negatives cluster gained H3K4me3	#Expected Negatives clusters at least one gained	Chi Square *P-value*
**Female**	5975	2987	2988	256	220.46	185	220.54	11.457 P-value <0.001
**Male**	6053	3023	3030	275	226.24	178	226.76	20.994 P-value <<0.001

Table S4b. In brain, Z- clusters, in comparison to Z+ clusters, are significantly enriched in depleted H3K4me3 peaks in human compared to chimps and macaques only in females.

Tissue	#Clusters	#Positives Cluster	#Negatives Negative	#Positives cluster depleted at least one H3K4me3	#Expected positives gained	#Negatives cluster depleted at least one H3K4me3	#Expected Negatives gained	Chi Square *P-value*
**Female**	5975	2987	2988	39	71.49	104	71.51	29.527 P-value <<0.001
**Male**	6053	3023	3030	62	66.92	72	67.08	0.722 p-value ~0.4

Table S5a. *P-values* of Mann-Whitney test comparing physical dimensions of Z+ and Z- clusters

Tissue	Median length of Z+ clusters (Male)	Median length of Z- clusters (Male)	Male *P-value*	Median length of Z+ clusters (Female)	Median length of Z- clusters (Female)	Female *P-value*
**Brain**	100813	81953	1.74E-06	90674	91422	0.86969
**Cerebellum**	90838	96469	0.15828	92790	102161	0.05200
**Kidney**	98079.5	115206	2.33E-07	83051.5	99106	0.00382
**Heart**	87304	115284	7.89E-07	91020	112959	2.91E-05
**Liver**	73028	106214	4.96E-17	NA	NA	NA
**Testis**	97548	106808	0.00039	NA	NA	NA

Table S5b. *P-values* of one-tailed Monte Carlo simulation to determine whether the difference observed between density of Z+ and Z- clusters could have happened by chance, as a function of the number of genes in a cluster. For this simulation, the number of Z+ and Z- genes are kept the same as observed and gene order is randomized. In each iteration, the number of occurrence of clusters of specified size is counted, if it is great or greater than the observed number of clusters of that size, Monte Carlo counter is incremented. Empirical P is then calculated after 1000 iterations.

Tissue/ Gender	*P-value* per Gene Cluster containing this number of genes												
	1	2	3	4	5	6	7	8	9	10	11	12	13
**Brain/M**	0.9980	1.0000	0.4376	0.9940	0.5554	0.6943	0.9301	0.8262	0.9031	0.6913	NA	NA	NA
**Cere./M**	1.0000	0.9950	0.0270	0.0130	0.0959	0.0090	0.0350	0.0230	0.0290	0.5504	0.0230	0.0819	NA
**Kidney/M**	0.9990	0.2527	0.0010	0.0010	0.0010	0.0010	0.0010	0.0010	0.0010	0.0160	0.0020	0.0010	0.0160
**Heart/M**	1.0000	0.3127	0.0010	0.0010	0.0010	0.0010	0.0010	0.0200	0.0010	0.0420	0.0010	0.0010	0.1748
**Liver/M**	0.1968	0.0010	0.0010	0.0010	0.0010	0.0030	0.0789	0.5724	0.0440	0.7592	0.2308	NA	NA
**Testis/M**	0.8511	0.5015	0.0310	0.0020	0.0010	0.0010	0.0010	0.0010	0.0010	0.0020	0.0020	0.0010	0.0010
**Brain/F**	0.8971	0.4525	0.4236	0.9530	0.8591	1.0000	0.3137	1.0000	0.4236	1.0000	NA	NA	NA
**Cere./F**	0.9980	0.9880	0.7493	0.0010	0.0999	0.0010	0.0420	0.2238	0.0310	0.1039	0.3267	NA	NA
**Kidney/F**	0.9970	0.2907	0.0010	0.3586	0.3117	0.5544	0.2807	0.1888	0.0559	0.7732	NA	NA	NA
**Heart/F**	1.0000	0.8561	0.1269	0.0060	0.0010	0.0100	0.0080	0.0020	0.0050	0.5954	0.2817	NA	NA

Cere. = Cerebellum
M = Male
F = Female

Table S6a. There are more strictly tissue-specifically up-regulated genes in Cerebellum and Liver than expected by chance. If we define strictly tissue specific up-regulated genes as the genes with Z score of more than one in one tissue and zscore of negative or zero in all other tissues and then estimate their number in each tissue based on the ratio of total number of strictly tissue specific up-regulated genes to the total number of expressed genes (across all tissues), and define expected number of strictly tissue specific genes by number of genes which are expressed in that tissue multiplied to this ratio, the observed number of tissue-specific genes in Cerebellum and Liver are significantly higher than the number expected by chance. Number of strictly tissue-specific genes is significantly below the expected number in Brain and Heart.

**Tissue**	**#Genes Expressed (RPKM>2)**	**#Strictly Tissue Specific Genes**	**Expected**	**Chi-Squared**	***P-value***
**Brain**	10020	7	29.3038	16.9759	<<0.01
**Cerebellum**	9400	45	27.4906	11.1522	<0.01
**Kidney**	9619	15	28.1311	6.1293	~0.02
**Heart**	9328	13	27.2800	7.4750	<0.01
**Liver**	8917	51	26.0780	23.8171	<<0.01
**Testis**	10845	39	31.7165	1.6726	~0.2

Table S6b. There are more tissue-specifically up-regulated genes in Cerebellum than expected by chance. By relaxing the definition of tissue-specific up-regulated genes to genes with Z>1 in one and only one tissue, more tissue-specifically up-regulated genes are detected. If we estimate number of tissue specifically up-regulated genes in each tissue based on the ratio of total number of tissue specifically up-regulated genes to the total number of expressed genes (across all tissues), and define expected number of tissue specific genes by number of genes which are expressed in that tissue multiplied to this ratio, the observed number of tissue-specific genes in Cerebellum is significantly higher than the number expected by chance. Number of tissue specific genes is significantly below the expected number in Brain and Heart.

**Tissue**	**#Genes Expressed (RPKM>2)**	**#Tissue Specific Genes**	**Expected**	**Chi-Squared**	***P-value***
**Brain**	10020	39	557.9786	482.7045	<<0.01
**Cerebellum**	9400	1230	523.4530	953.6838	<<0.01
**Kidney**	9619	524	535.6484	0.2533	~0.6
**Heart**	9328	365	519.4436	45.9199	<<0.01
**Liver**	8917	457	496.5564	3.1511	~0.07
**Testis**	10845	622	603.9200	0.5413	~0.55

Table S6c. If a focal tissue-specific up-regulated gene has a neighbor closer than 100Kb, overall this neighbor is more likely to be up-regulated. Binomial test between the number of tissue-specific up-regulated genes with at least a neighbor in ±100Kb to the number of these genes whose closest neighbor in 100Kb is also a Z+ gene, Chi-Squared = 68.03, p-value<<0.01. Overall 59.45% of these genes have a Z+ gene as their closest neighbor. Expected is the expected number of tissue specifically up-regulated genes with a neighbour up-regulated in the same tissue. Tissue-specific up-regulated genes are defined as explained in table S6b.

Tissue	#TSU* with a neighbor in ±100Kb	#TSU* with Z+ neighbor in ±100Kb	%with Z+ neighbor in ±100Kb	Expected Value	Binomial *P-value*
**Brain**	32	19	59.375	15.731	0.2902
**Cerebellum**	1076	606	56.3197	537.9587	3.33E-05
**Kidney**	501	348	69.4611	250.4808	1.16E-18
**Heart**	336	196	58.3333	167.9871	0.0022
**Liver**	413	220	53.2688	206.4841	0.1844
**Testis**	547	338	61.7916	273.479	3.09E-08

* TSU: Tissue-Specific Up-regulated genes

Table S6d. If a focal tissue-specific up-regulated gene lacks a neighbor closer than 100Kb, overall its closest neighbor is less likely to be a Z+ gene. Binomial test between the number of tissue-specific up-regulated genes without any neighbor in ±100Kb to the number of these genes whose closest neighbor is a Z+ gene. Expected value for the number of tissue-specific up-regulated genes having a Z+ as their closest neighbor is calculated by multiplying number of tissue-specific up-regulated genes to probability of a gene being Z+ in the corresponding tissue, Chi-Squared = 12.43, p-value<0.04. Overall 39.46% of these genes were observed to have a Z+ gene as their closest neighbor. Tissue-specific up-regulated genes are defined as explained in table S6b.

Tissue	#TSU* without a neighbor in 100Kb	#TSU* with Z+ as its closest neighbor	%with Z+ closest neighbor	Expected Value	Binomial *P-value*
**Brain**	7	4	57.1429	3.4412	0.7222
**Cerebellum**	154	57	37.013	76.9941	0.0016
**Kidney**	23	14	60.8696	11.4991	0.3074
**Heart**	29	13	44.8276	14.4989	0.7111
**Liver**	44	21	47.7273	21.9983	0.8804
**Testis**	75	22	29.3333	37.4971	0.0004

* TSU: Tissue-Specific Up-regulated genes

Table S6e. Genes with Tissue-specific upregulation are not clustered. The number of tissue-specific up-regulated (TSU) genes with a TSU downstream neighbor in the same tissue is shown. *P-value* of one-tailed Monte Carlo simulation is also reported. For this simulation the number of TSU genes are kept the same as observed in corresponding tissue but their gene order is randomized by shuffling all the genes including TSU ones in each tissue to ask if similar number of TSU downstream neighbors could have happened just by chance under the null of random gene order. This is repeated for 10,000 times and the number of TSU genes with a TSU downstream neighbor, regardless of their distance to the focal gene, is counted; If equal or greater than the observed number of genes with a TSU downstream neighbor, Monte Carlo counter is incremented and overall P-value calculated. We also show, for information, the proportion of TSU pairs where they are within 100kb of each other. Tissue-specific up-regulated genes are defined as explained in table S6b.

Tissue	#TSU* genes	#Downstream neighbour is also TSU in the same tissue	%Within 100Kb	Monte Carlo Mean (SD)	Monte Carlo P-value
**Brain**	39	0	0	0.1135 (0.3347)	1
**Cerebellum**	1230	128	77.343	116.0135 (9.661)	0.11979
**Kidney**	524	28	92.857	21.056 (4.407)	0.07629
**Heart**	365	13	92.307	10.1909 (3.082)	0.22058
**Liver**	457	20	85	16.0068 (3.865)	0.17988
**Testis**	622	0	0	29.5799 (5.112)	1

* TSU: Tissue-Specific Up-regulated

Table S7. Spearman correlation between female and mean of male Z scores per tissues, without removing zero Z scores

Tissue	*Rho*	*P-value*
**Brain**	0.52967	<<0.0001
**Cerebellum**	0.32532	9.01e-319
**Heart**	0.45401	<2.2e-16
**Kidney**	0.43073	<2.2e-16

Tables S8a. Spearman correlation between standard residual of standard major axis estimation between Z of male and female for a focal gene and residual of its nearest downstream neighbor, without removing zero Z scores

Tissue	Non-overlapping *P-value*	Non-overlapping *Rho*	Overlapping *P-value*	Overlapping *Rho*
**Brain**	0.00018	0.03994	0.00325	0.10408
**Cerebellum**	0.03109	0.02304	9.10E-06	0.15636
**Heart**	1.42E-05	0.04638	8.04E-05	0.13913
**Kidney**	6.95E-19	0.09465	0.01206	0.08883

Table S8b. Spearman correlation between standard residual of standard major axis estimation between Z of male and female for a focal gene and mean of residuals of its two nearest neighbors, without removing zero Z scores

Tissue	Non-overlapping *P-value*	Non-overlapping *Rho*	Overlapping *P-value*	Overlapping *Rho*
**Brain**	5.71E-06	0.05461	0.00613	0.09694
**Cerebellum**	0.002448527	0.03648	0.00076	0.11884
**Heart**	5.86E-09	0.07003	0.00019	0.13129
**Kidney**	3.24E-23	0.11913	0.00018	0.13190

Table S8c. Spearman correlation between standard residual of focal gene and the mean of standard of residual of all neighbors within 100kb of the focal gene, without removing zero Z scores

Tissue	Spearman *P-value*	Spearman *Rho*
**Brain**	4.00E-08	0.04816
**Cerebellum**	0.00847	0.02310
**Kidney**	1.71E-39	0.11504
**Heart**	1.87E-05	0.03755

Table S9. No evidence for X chromosome enrichment in sex biased genes. If top 5% of the genes are selected based on their standard residual to standard major axis estimation between Z of male and female, no enrichment of x linked genes is observed compared to autosomal genes. Brawand's dataset includes 466 genes on chromosome X and 12561 genes on autosomes. Note, genes with Z=0 are not excluded from this analysis so expected number of genes are the same across different tissues.

	**Observed X**	**Expected X**	**Observed Autosome**	**Expected Autosome**	**Chi Squared**	***P-value***
**Brain**	21	23.3	628	628.05	0.2270	>0.6
**Cerebellum**	17	23.3	632	628.05	1.7283	>0.15
**Heart**	26	23.3	623	628.05	0.3535	>0.5
**Kidney**	21	23.3	628	628.05	0.2270	>0.6
